# Brain Activation During Active Balancing and Its Behavioral Relevance in Younger and Older Adults: A Functional Near-Infrared Spectroscopy (fNIRS) Study

**DOI:** 10.3389/fnagi.2022.828474

**Published:** 2022-03-25

**Authors:** Nico Lehmann, Yves-Alain Kuhn, Martin Keller, Norman Aye, Fabian Herold, Bogdan Draganski, Wolfgang Taube, Marco Taubert

**Affiliations:** ^1^Department of Sport Science, Institute III, Faculty of Humanities, Otto von Guericke University, Magdeburg, Germany; ^2^Department of Neurology, Max Planck Institute for Human Cognitive and Brain Sciences, Leipzig, Germany; ^3^Department of Neurosciences and Movement Science, Faculty of Science and Medicine, University of Fribourg, Fribourg, Switzerland; ^4^Department of Sport, Exercise and Health, University of Basel, Basel, Switzerland; ^5^Research Group Degenerative and Chronic Diseases, Movement, Faculty of Health Sciences Brandenburg, University of Potsdam, Potsdam, Germany; ^6^Laboratory for Research in Neuroimaging, Department of Clinical Neuroscience, Lausanne University Hospital and University of Lausanne, Lausanne, Switzerland; ^7^Center for Behavioral Brain Science, Otto von Guericke University, Magdeburg, Germany

**Keywords:** aging, neuroimaging, functional near-infrared spectroscopy (fNIRS), balance, postural control, prefrontal cortex, neural inefficiency

## Abstract

Age-related deterioration of balance control is widely regarded as an important phenomenon influencing quality of life and longevity, such that a more comprehensive understanding of the neural mechanisms underlying this process is warranted. Specifically, previous studies have reported that older adults typically show higher neural activity during balancing as compared to younger counterparts, but the implications of this finding on balance performance remain largely unclear. Using functional near-infrared spectroscopy (fNIRS), differences in the cortical control of balance between healthy younger (*n* = 27) and older (*n* = 35) adults were explored. More specifically, the association between cortical functional activity and balance performance across and within age groups was investigated. To this end, we measured hemodynamic responses (i.e., changes in oxygenated and deoxygenated hemoglobin) while participants balanced on an unstable device. As criterion variables for brain-behavior-correlations, we also assessed postural sway while standing on a free-swinging platform and while balancing on wobble boards with different levels of difficulty. We found that older compared to younger participants had higher activity in prefrontal and lower activity in postcentral regions. Subsequent robust regression analyses revealed that lower prefrontal brain activity was related to improved balance performance across age groups, indicating that higher activity of the prefrontal cortex during balancing reflects neural inefficiency. We also present evidence supporting that age serves as a moderator in the relationship between brain activity and balance, i.e., cortical hemodynamics generally appears to be a more important predictor of balance performance in the older than in the younger. Strikingly, we found that age differences in balance performance are mediated by balancing-induced activation of the superior frontal gyrus, thus suggesting that differential activation of this region reflects a mechanism involved in the aging process of the neural control of balance. Our study suggests that differences in functional brain activity between age groups are not a mere by-product of aging, but instead of direct behavioral relevance for balance performance. Potential implications of these findings in terms of early detection of fall-prone individuals and intervention strategies targeting balance and healthy aging are discussed.

## Introduction

It is generally accepted that the ability to produce well-coordinated, smooth and accurate movements decreases with aging (Seidler et al., 2010; Papegaaij et al., 2014; Hunter et al., 2016). One example for the decline in motor control over the lifespan is the development of deficits in balance and gait, which are associated with an increased frequency of slips, stumbles and falls with advancing age (Delbaere et al., 2010; Ambrose et al., 2013). Injurious falls bear not only the potential of dramatic consequences for the individual by limiting mobility and physical autonomy and thus, contributing to morbidity and mortality (Tinetti and Williams, 1998; Kannus et al., 1999), but have also considerable socioeconomic implications, for instance in terms of healthcare spending (Florence et al., 2018). It is against this background that effective neural control strategies of balance in the younger and the older need to be characterized. Knowledge of neurobiological mechanisms linked to balance performance does not only allow to identify fall-prone individuals, but also constitutes the basis for developing targeted preventive and rehabilitative intervention strategies (Seidler et al., 2010; Granacher et al., 2011; Papegaaij et al., 2014; Seals et al., 2016).

There is general agreement that the age-related degeneration of balance control originates from a complex, multifactorial interplay of changes at the pre- and postsynaptic sides of the neuromuscular system (Seidler et al., 2010; Papegaaij et al., 2014; Hunter et al., 2016). With respect to the brain, it has been recognized that the interrelation and information transfer between cortico-cortical and cortico-subcortical loops subserving balance control changes as a function of age (Papegaaij et al., 2014; Takakusaki, 2017; Dijkstra et al., 2020), which is probably related to the shift from automatic to more controlled processing in postural control (Clark, 2015; Richer and Lajoie, 2020). Although challenging from a methodological perspective (Perrey and Besson, 2018; Menant et al., 2020), studies using functional neuroimaging methods like electroencephalography (EEG) or near-infrared spectroscopy (fNIRS) in freely moving participants made important contributions to our understanding of neural aging processes related to balance and gait. One of the key findings of these studies is that older, compared to younger adults, commonly exhibit an increase in neural activation during balancing, particularly in prefrontal brain areas (Lin et al., 2017; Rosso et al., 2017; Teo et al., 2018; St George et al., 2021; but see also Marusic et al., 2019). This increased brain activity during balancing may manifest itself in cortical overactivation, but also in more widespread (neural dedifferentiation) or less lateralized patterns of activity (see Seidler et al., 2010; Herold et al., 2017b; Li et al., 2018; Fettrow et al., 2021; Yeung and Chan, 2021, for reviews). Of note, similar aging-associated changes in the patterns of brain activity have also been predicted by theoretical accounts on neurocognitive aging (Festini et al., 2018; Yeung and Chan, 2021, for reviews), and the prevailing view is that these changes reflect a more limited information processing capacity of the aging brain. Specifically, it is assumed that in the older more cortical involvement is required to cope with given task demands, in a situation of increased competition for limited neural resources (Seidler et al., 2010; Granacher et al., 2011; Papegaaij et al., 2014; Fettrow et al., 2021). A better understanding of the mechanisms underlying balance control in aging would provide a solid base for the development and application of focused physical and non-physical intervention approaches to enhance balance performance.

Despite the presence of robust age differences in the neural control of balance, the implications of these findings in terms of the age-related degradation in balance control are not well understood. For instance, it would be of utmost importance to identify brain regions whose balance-induced activation is not only related to age, but also to balance performance. Brain regions that fulfill both of these criteria would qualify as putative *mediators* in the relationship between aging and balance performance (James and Brett, 1984). While the implicit assumption in the above scenario is that a given brain area is behaviorally relevant across age groups, it is also possible that the process of aging is associated with a heightened (lowered) reliance on certain cortical areas during balancing. This reliance could be a result of a different brain reserve in younger and older people (Li et al., 2018; Menant et al., 2020; Fettrow et al., 2021); consequently, effective postural control strategies might vary as a function of age. Here, age would serve as a *moderator* in the relationship between functional brain activity and balance performance. Collectively, more light on the question whether age-related differences in brain activity during balancing reflect a functional (i.e., associated with good performance) or a dysfunctional (i.e., associated with worse performance) phenomenon (Fettrow et al., 2021; Yeung and Chan, 2021) can only be shed when studying both neural activation during balancing and its relationship to balance performance.

The present paper aims to contribute to a more comprehensive understanding of the cortical dynamics subserving balance performance in healthy younger and older adults. Specifically, we use fNIRS, an optical brain imaging method that detects cortical activation by measuring task-evoked changes of oxygenated and deoxygenated hemoglobin (Villringer and Chance, 1997), to examine cortical hemodynamics during bipedal standing on a wobble board. As postural sway in standing is more strongly related to age than measures derived from gait analysis (Park et al., 2016), tasks requiring to maintain a safe standing position seem to be particularly well suited for studying the neurobiological mechanisms underlying balance control in the older. Here, we first compared whether cortical hemodynamic responses during balancing would differ between older and younger adults in brain areas known to be involved in postural control. In the older, we expected to see overactivation compared to younger individuals in brain regions associated with higher-order control processes, especially in prefrontal cortical areas (Seidler et al., 2010; Herold et al., 2017b; Yeung and Chan, 2021). Younger individuals, on the contrary, were hypothesized to show higher activation than older participants in brain areas related to task automaticity, such as the sensorimotor cortex (Seidler et al., 2010; Herold et al., 2017b). To address the functional relevance of the former findings, we then related functional brain activation to balance performance outcomes. To this end, we used robust regression models to identify task-relevant brain areas and to check for a potential mediating role of age in the hemodynamics-performance relationship. All of the abovementioned analyses were conducted on sufficiently reliable hemodynamics data, as suggested in the literature on functional neuroimaging (Menant et al., 2020; Yücel et al., 2021).

## Materials and Methods

### Participants and Experimental Design

Data from 35 older (age range 65–80 years) and 27 younger adults (age range 20–33 years) of both sexes free from neurological (including cerebral lesions), movement or psychiatric disorders, heart disease (incl. cardiac pacemakers), epilepsy, diabetes, major vision problems, and diagnosed dementia were considered in the present study (for group characteristics, see Table 1). Participants were right-handed and reported no history of falls. All participants were part of a randomized controlled trial on neural adaptations in response to long-term balance training, and most responded to community advertisements. The study was performed in accordance with the ethical standards as laid down in the 1964 Declaration of Helsinki and its later amendments. Approval was granted by the *Commission cantonale d’Éthique de la Recherche sur l’être humain Vaud* (CER-VD 2016-00738, 13/06/2016). The study protocol was prospectively registered in the *swissethics* clinical trial register (date of full registration: 12/07/2016). Written informed consent was obtained from all participants.

**TABLE 1:** Between-group comparison of demographic and physical activity data.

	Older	Younger	Older vs. Younger
Number of participants	35	27	χ^2^(1) = 2.06, *p* = 0.15
Age (years)	70.14 (4.05)	24.78 (3.48)	*U*(35,27) = 945, *p* < 0.001
Sex (♂\♀)	17/18	18/9	χ^2^(1) = 2.03, *p* = 0.15
Sport score (Baecke et al., 1982; Pols et al., 1995)	3.16 (0.61)	3.44 (0.64)	*t*(54.58) = –1.73, *p* = 0.09

*Descriptive statistics refer to frequencies (in the case of χ^2^ test), median and interquartile range (in case of Mann–Whitney U test) or mean and SD (in case of Welch’s t-test).*

To address our research questions, we used a cross-sectional design where each participant’s testing took place during one measurement session. The session started with behavioral balance assessment on a free-swinging platform (Posturomed) and wobble boards with different levels of difficulty, as outlined below. Afterward, fNIRS data was recorded while participants balanced on a wobble board. Several other behavioral, fNIRS, and TMS assessments were also conducted during this session. These tests are not discussed further since they are not of relevance for the present study. Immediately before the balance and fNIRS tests in the lab, all subjects underwent cognitive assessment consisting of working memory (“n-back” procedure, Gevins and Cutillo, 1993), long-term memory (Test of Visual and Verbal Memory Retention [VVM], Schellig and Schächtele, 2009), and attention tests (revised d2 Test of Attention [d2-R], Brickenkamp et al., 2010).

After an initial data quality check, we analyzed (1) age-group differences in functional brain activity during balancing and (2) correlated functional brain activity with balance performance (regression/moderated regression). In case of age-specific regional activity differences together with a correlation between hemodynamics and balance performance in the same area, we also (3) tested for the presence of statistical mediation.

### Behavioral Assessment

To investigate relationships between cortical hemodynamics and balance ability, two established balance tasks were used (Taube et al., 2008; Wälchli et al., 2018), as outlined in the following. Before these tests were conducted, participants were allowed to familiarize with the devices and tasks.

First, participants were asked to stand on a movable platform (Posturomed, Haider Bioswing, Pullenreuth, Germany) reliably measuring postural sway in the transversal plane (Boeer et al., 2010). Four balance conditions were chosen based on common use in the literature: regular standing with eyes open (RS-EO), regular standing with eyes closed (RS-EC), single-leg standing with eyes open (SLS-EO), and single-leg standing with eyes closed (SLS-EC) (Kapteyn et al., 1983). Participants were instructed to stand upright, with their hands on their hips, and remain as still as possible for the duration of the trial. In case that participants’ movement execution deviated from these instructions, the respective trial was not considered in the statistical analyses. For each condition, three consecutive trials with a length of 30s each were conducted. Reflecting markers were fixed on the movable platform to sample data at 120 Hz with a Vicon 512 system (Vicon Motion Systems Ltd, Oxford, United Kingdom). Data were analyzed offline with MATLAB. Specifically, trajectories of the markers were filtered (low-pass Butterworth) and then the total marker displacement (displacement of the platform) was calculated. For all conditions, the best trial was used for further analysis. The conditions SLS-EO and SLS-EC were dropped from statistical analyses due to many missing data in the older participants.

Second, we recorded the displacements of the center of pressure (CoP) in upright bipedal standing during balancing on a wobble board. Four commercially available wobble boards with different heights and areas of support were used. All measurements took place on a triaxial force plate with a sampling rate of 4000 Hz (OR6-7-2000 force platform, Advanced Mechanical Technology Inc., Watertown, MA, United States). Participants received the same instruction as described above for the free-swinging platform, and only error-free trials were analyzed statistically. Consequently, we only considered the data from the two easiest levels (level 1: 41.5 cm diameter, 8 cm height; level 2: 36.5 cm diameter, 8.5 cm height) for further data analyses, because most older participants were not able to accomplish the more difficult conditions. For each wobble board level a participant was able to master, two trials with a length of 30s each were conducted. MATLAB-based preprocessing included a correction for the height of the board and filtering of the signal (low-pass Butterworth). Afterward, the CoP time series on the antero-posterior as well as the medio-lateral axes were calculated. For subsequent analyses, we used the total path length of the CoP as derived from the statokinesigram. Only the best trial (lowest path length) of a participant for each wobble board was considered for statistical analyses.

We also computed a latent variable for balance performance based on the scores of all bipedal balancing tasks (i.e., wobble board levels 1 and 2, RS-EO, RS-EC). Such a factor score allows to indirectly determine the potential presence of hemodynamic activity that is functionally relevant over a range of balance tasks. The appropriateness of the use of principal components analysis (PCA) was tested using the Kaiser–Meyer–Olkin (KMO) measure for sampling adequacy (Kaiser and Rice, 1974), Bartlett’s (1954) test for assessing the intercorrelations of scores, and the determinant of the correlation matrix as a measure of multicollinearity (Field et al., 2012). As recommended in the literature (Schmitt, 2011), the number of factors to retain was determined using Horn’s (1965) parallel analysis.

**FIGURE 1:**
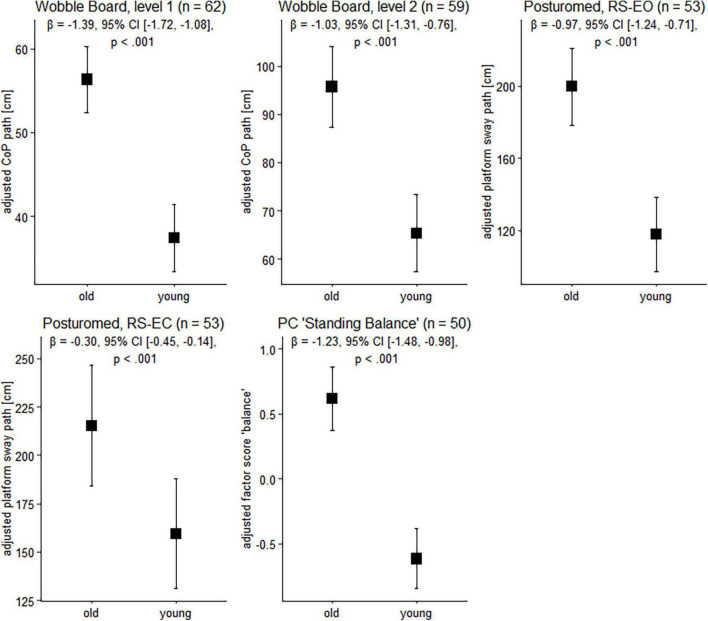
Balance performance in older and younger participants. Data are presented as predicted group means (corrected for the influence of sex and Baecke’s sport score) and the corresponding 95% CI (error bars). Note that the beta-coefficients reflecting the between-group differences in units of standard deviation are conceptually similar to Cohen’s *d*. Rules of thumb for interpreting this effect size are 0.2 < | *β*| < 0.5 “small,” 0.5 < | *β*| < 0.8 “medium,” and | *β*| > 0.8 “large” effects, respectively (cf. Cohen, 1988).

We exclusively used wobble board balance performance (level 1) as a dependent variable in later brain-behavior correlations. This choice was made because wobble board performance had a higher number of eligible cases compared to all other behavioral tests (including the factor score; see Figure 1). Furthermore, wobble board performance (level 1) loaded highly on the latent variable (see Table 2) and is therefore strongly correlated with the other balance tests. Therefore, focusing on this behavioral variable avoids redundancy in data analyses.

**TABLE 2:** Correlations (factor loadings) between the original variables and the extracted component “bipedal standing balance” based on *n* = 50 eligible cases.

	Wobble board, level 1	Wobble board, level 2	Posturomed, RS-EO	Posturomed, RS-EC
Factor loading	0.88	0.93	0.82	0.81

### Functional Near-Infrared Spectroscopy Experimental Setup

**FIGURE 2:**
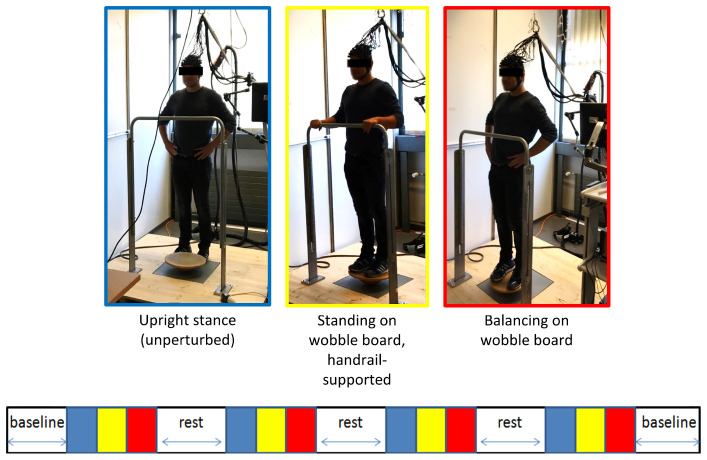
Schematic overview of the fNIRS measurement. A block design comprising of three conditions à 30 s each was used to study the cortical activation during balancing (see main text). Stimulus event onsets are shown in the lower part of the figure. Blue, unperturbed upright stand (low-level baseline); yellow, standing on the wobble board (handrail-supported; active control condition); red, balancing on the wobble board. The blocks were interspersed by short (≈ 30 s−1 min) rest periods (white). Baseline measurements during quite standing were conducted before and after the experiment to assess long-term drift in the fNIRS signal. Please note that the NIRS-overcap mentioned in the main text is not depicted here.

Hemodynamic response alterations during balancing were assessed using the stationary NIRScout continuous-wave imaging system (NIRx Medical Technologies, Glen Head, NY, United States) with silicon photodiodes as detectors and operating with simultaneous frequency-encoded dual-wavelength illumination at 760 and 850 nm. Parallel source firing allowed for a scan rate of 7.81 Hz, whereas the minimum distance of simultaneously illuminated sources was set to 9 cm to rule out optical crosstalk. Optodes were attached to a stretchy NIRScap with pre-cut slits corresponding to international EEG 10-5 coordinates (Oostenveld and Praamstra, 2001) by means of spring-loaded grommets, therefore allowing for consistent landmarking of optode locations between participants (Menant et al., 2020). The NIRScap was available in two different sizes (56 and 58 cm head circumference) to ensure an appropriate fit to the participants’ heads. Before the optodes were fixed, hair was brushed away within every hole of the cap to ensure a good light coupling. An overcap produced by NIRx was used to avoid interference of ambience light during the experiment. To ensure as stable positioning as possible of the head probe during balancing, we used an elastic string (with a tied carabiner) affixed on the ceiling of the room to relieve source and detector cables from mechanical strain (Figure 2). Before putting on the overcap, all source and detector cables were arranged in a way that they converged in the back of the head/neck. From there they exited the overcap and went upwards to the carabiner. As a last step before the start of the measurement, an automatic calibration was performed to determine an appropriate amplification factor for each source-detector combination and to judge the signal quality. Recording was only started if each channel in the signal quality map (generated by the NIRStar software) was labeled as “excellent” or at least “acceptable.”

During the fNIRS recording, all participants engaged in a fixed sequence of conditions for four times (block design), and blocks were interspersed by short (≈ 30 s-1 min) rest periods to allow the balancing-induced hemodynamic responses to return toward baseline levels (see Figure 2). In the first condition, participants stood still on the ground (i.e., without the wobble board) for 30 s with the hands resting in their hips. Second, participants were asked to unhurriedly grasp the handrail in front of them and to get on the wobble board (level 1). When participants assumed this position, a 30 s period of relaxed, handrail-supported standing started after manual triggering of the experimenter. This active control condition was chosen as the reference for later calculation of the hemodynamic activity during balancing. Third, a visual countdown was presented to participants signalizing the start of wobble board balancing for 30 s. After the countdown lapsed, participants were instructed to move their hands to their hips and to remain as still as possible for the duration of the trial. The standardized positioning of the hands was chosen to minimize unsystematic effects related to arm movements in the imaging signal. Note that we used the easiest wobble board for both age groups to ensure that a direct comparison of hemodynamic activity can be made whilst avoiding that a high number of cases must be excluded due to difficulties with mastering the wobble board and/or excessive head motion (Menant et al., 2020). We did not register any error (e.g., releasing the hands from the hips, grabbing the handrail or stepping down with the feet) during fNIRS-balancing recordings in neither age group.

During each of the abovementioned conditions, participants’ attention was directed to a fixation cross shown on a computer screen in front of them. Furthermore, participants were asked to refrain from speaking, laughing, and from executing movements that were not explicitly instructed (e.g., outstretching the arms).

### Functional Near-Infrared Spectroscopy Probe Design and Regions of Interest

**FIGURE 3:**
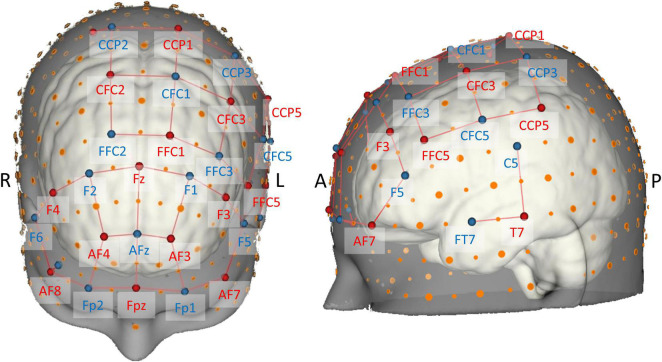
Brain map of the fNIRS head probe covering prefrontal, motor, and temporal regions. International EEG 10-5 coordinates (Oostenveld and Praamstra, 2001) are depicted next to source (red) and detector (blue) optodes. Note the short-distance channels near 10-5 positions AF8 and CCP2.

The probe consisted of 16 sources and 16 detectors resulting in a total of 38 long-separation channels from which local changes in hemodynamic responses were measured. Stabilizing links were used to fix source–detector distances of long-separation channels to approximately 30 mm, therefore ensuring reasonable sensitivity to the cortex and a good signal-to-noise ratio (Strangman et al., 2013; Brigadoi and Cooper, 2015). The probe configuration also contained two short-separation channels near 10-5 positions AF8 and CCP2 (Figure 3) with a source–detector separation of ≈13 mm (Brigadoi and Cooper, 2015), which were used for nuisance regression later on (Gagnon et al., 2011; Tachtsidis and Scholkmann, 2016).

The probe geometry was designed using the NIRSite software and was set up to cover important superficial cortical areas known to be related to postural control and balance (cf. Seidler et al., 2010; Herold et al., 2017b; Dijkstra et al., 2020; Yeung and Chan, 2021). To image bilateral prefrontal cortex, we adapted the “16 × 16 prefrontal-motor” configuration as provided by NIRx. The arrangement of the motor channels was inspired by previous studies (Kurz et al., 2012; Herold et al., 2017a), but was slightly modified in order to be more sensitive to activity in Brodmann area 6 (SMA). Furthermore, due to the limited number of available optodes, we opted for a left-lateralized motor probe geometry (Rosso et al., 2017), especially to be able to measure activity in ventral motor areas. Finally, cortical activity of the left superior temporal gyrus (Karim et al., 2012, 2014; Rosso et al., 2017) was measured with two channels grouped around position T7 according to the EEG 10-5 system. Theory-derived channel locations were validated in terms of adequate spatial positioning by mapping each optode’s 10-5 location onto the probabilistic automated anatomical labeling atlas ([aal2]; Tzourio-Mazoyer et al., 2002; Rolls et al., 2015) as implemented in the fNIRS Optodes’ Location Decider ([fOLD]; Zimeo Morais et al., 2018).

### Functional Near-Infrared Spectroscopy Data Processing and Reproducibility Analysis

Functional near-infrared spectroscopy data were preprocessed using Homer v2.8 (Huppert et al., 2009), an open-source, Matlab-based software package. All of the steps listed below were in line with the latest fNIRS data processing recommendations (Herold et al., 2018; Pinti et al., 2018; Menant et al., 2020).

First, noisy channels with a very low optical intensity (coefficient of variation > 10%) were excluded (enPruneChannels). This applied to 4.41% (SD 9.16%) of channels on average. The remaining raw optical intensity data was then converted to changes in optical density (OD) (hmrIntensity2OD). Next, motion artifacts were corrected using a wavelet transform [hmrMotionCorrectWavelet according to Molavi and Dumont (2012) with the interquartile range parameter set to 1.219 (Herold et al., 2019; Seidel et al., 2019; Carius et al., 2020)]. The signal was then bandpass filtered (hmrBandpassFilt) with a high-pass cut-off frequency of 0.01 Hz to account for instrumental noise and low frequency drifts, and with a low-pass cut-off frequency of 0.09 Hz (third-order Butterworth filter) to remove effects of spontaneously generated physiological components (Mayer waves: ≈0.1 Hz, heart rate: 1.6–1.8 Hz, breathing: 0.2–0.3 Hz) (Hwang et al., 2016; Pinti et al., 2018). Preprocessed optical density data of both wavelengths were then converted to oxygenated (oxyHb) and deoxygenated hemoglobin (deoxyHb) concentration changes using the modified Beer-Lambert equation (Brigadoi et al., 2014). An age- and wavelength-adjusted differential pathlength factor (DPF) required for this equation was calculated according to Scholkmann and Wolf (2013). Note that an individual DPF was calculated for every single participant. The hemodynamic response function (HRF) was deconvolved using a General Linear Model (GLM) based on ordinary least-squares regression (Ye et al., 2009) over the time period –5 to 25 s (–5 to 0: handrail-supported standing baseline, 0–25: balancing) (hmrDeconvHRF_DriftSS). The HRF was modeled using a consecutive sequence of Gaussian HRF basis functions with a standard deviation of 0.5 s and their means separated by 0.5 s, and a third order polynomial was included to account for baseline drift. Furthermore, to correct for systemic, non-cortical changes in scalp blood flow (Gagnon et al., 2011; Tachtsidis and Scholkmann, 2016), the short-separation fNIRS signal with the greatest correlation with the long-separation fNIRS channel in question was added as physiological regressor to the GLM. The recovered HRFs for each channel and chromophore (oxyHb and deoxyHb) were baseline corrected by subtracting the mean of the HRF between –5 and 0 s from each data point of the HRF.

Finally, for each channel and chromophore (oxyHb and deoxyHb), we calculated two indices derived from the baseline corrected HRF. We averaged oxyHb and deoxyHb concentrations across the four trial intervals to get the mean activation values throughout balancing (block average). Additionally, we fitted the HRFs of both chromophores with linear regression in order to estimate the slope coefficient (Mandrick et al., 2013), which might be sensitive regarding additional age group-distinguishing hemodynamic features. As with mean activation data, slope coefficients were subsequently averaged across task intervals. The dependent variables (block averages and slope coefficients) were calculated based on hemodynamics data in a time window from 0 s (stimulus onset) to 20 s (Takakura et al., 2015; Fujita et al., 2016; Herold et al., 2017a).

As commonly done in the field (Herold et al., 2017b), we applied within-participant averaging across channels belonging to a certain region of interest (ROI). To this end, we again used the aal2 atlas (Tzourio-Mazoyer et al., 2002; Rolls et al., 2015) as implemented in fOLD (Zimeo Morais et al., 2018). Based on the highest probability of a given channel to be part of a particular ROI, the channel was uniquely assigned to that ROI (Table 3). Channels of artifact as classified by Homer2 were discarded from ROI averaging and further analyses (Willems and Cristia, 2018).

**TABLE 3:** Assignment of channels (i.e., source-detector pairs) in the head probe to aal2 atlas regions (Tzourio-Mazoyer et al., 2002; Rolls et al., 2015) based on maximum probabilities as derived from fOLD (Zimeo Morais et al., 2018).

aal2 atlas region	Optodes (10-5 location)
Superior frontal gyrus, dorsolateral (SFG)	AF3_Fp1, AF3_F1, FFC1_FFC3, AF3_AFz, CFC3_CFC1, Fpz_Fp1, AF4_Fp2, AF4_F2, Fpz_Fp2, AF4_AFz
Superior frontal gyrus, medial (SFGmedial)	Fz_AFz, Fpz_AFz, Fz_F1, Fz_F2, FFC1_FFC2
Middle frontal gyrus (MFG)	FFC5_FFC3, CFC3_FFC3, S1_D1, S1_F1, AF7_D1, AF7_Fp1, S7_F6, S7_F2, AF8_F6, AF8_Fp2
Supplementary motor area (SMA)	FFC1_CFC1, CFC2_FFC2, CFC2_CFC1
Precentral gyrus (PreCG)	CFC3_CFC5, CFC3_CCP3, FFC5_CFC5, CFC2_CCP2, CCP1_CFC1
Postcentral gyrus (PoCG)	CCP5_CFC5, CCP1_CCP3, CCP5_CCP3
Middle temporal gyrus (MTG)	T7_FT7, T7_C5

As a last step before statistical analyses, we used the data from the subset of the younger (*n* = 15) and older participants (*n* = 17) that were assigned to the control group of the parent experimental study (see above) and assessed the test-retest reliability of the active test condition fNIRS data (measurements separated by 8 weeks). This procedure was chosen because there is now increasing awareness that the use of insufficiently reliable data is one of the major factors contributing to the reproducibility crisis in science in general (Loken and Gelman, 2017) and in functional neuroimaging in particular (Zuo et al., 2019; Menant et al., 2020). Regarding fNIRS measurements of whole-body movements, this issue is further aggravated by changes in heart rate and blood pressure, potential displacements of the optodes, and other alterations of the signal like head positioning (e.g., cerebral blood flow when looking down vs. looking upwards; see Vitorio et al., 2017; Menant et al., 2020). Benchmark values from previous reliability studies are of limited value for the present investigation, because these studies used portable fNIRS devices and they exclusively analyzed walking (Stuart et al., 2019; Broscheid et al., 2020).

Regions of interest-wise reliability was assessed using the two-way mixed model intraclass correlation for average measurements, where agreement was defined in terms of consistency. According to the McGraw and Wong (1996) convention, this type of model is termed ICC(A,k). With four calculated fNIRS parameters (HbO_BA, HbO_SC, HbR_BA, and HbR_SC) and seven ROIs, a total of 28 variables are present. In 14 variables insufficient ICCs (<0.4 according to Fleiss, 1986; Cicchetti, 1994) were detected (see Supplementary Table 1), and these variables were excluded from further analyses. Based on this criterium, the ROI MTG had to be excluded completely.

### Statistical Analyses

Between-group comparisons were conducted dependent on the level of measurement and whether assumptions of the *t*-test were met. Therefore, results are reported as chi square, Mann–Whitney, or *t*-statistics (Welch’s *t*-test). If not otherwise stated, statistical tests of significance carried out throughout the manuscript were performed two-sided.

In fNIRS imaging, the consistency of signals between sessions even within the same person can be compromised by factors like body and head movement or changes in heart rate and blood pressure (Menant et al., 2020). For these and other reasons, the assumptions of standard statistical tests relying on the general linear model are difficult to fulfill in functional neuroimaging (Poline and Brett, 2012; Seidel et al., 2019). Therefore, to address the main research questions in this paper, we use statistical methods insensitive to outliers and not relying on homoscedastic and independent errors and normality (Maronna et al., 2006; Field and Wilcox, 2017). Specifically, robust linear models (ANCOVA- and regression-type) as described in the following were analyzed based on an MM-type estimator (Yohai, 1987; Koller and Stahel, 2017) using iteratively reweighted least squares estimation as implemented in the robustbase v0.93-6 package (Maechler et al., 2020) running in R v3.6.1 (R Development Core Team, 2018). Standard errors (and associated test statistics and *p*-values) and confidence intervals were estimated based on *B* = 10000 bootstrap replications (Salibián-Barrera et al., 2008). Unless otherwise stated, robust linear models were adjusted for biological sex and Baecke’s sport score (Baecke et al., 1982; Pols et al., 1995). Multiple testing correction based on the false discovery rate (FDR; Benjamini and Hochberg, 1995) was applied to *p*-values obtained from statistical tests on fNIRS data.

First, we analyzed whether balance performance would differ among age groups (equation 1). Note that the values for balance performance were standardized (*z*-transformed) before model estimation, such that the regression coefficient of age group corresponds to the between-group difference in units of pooled standard deviation and is thus, conceptually similar to the effect size *d* (Cohen, 1988).


(1) Performance(z-transformed)i=b0+b1agegroup+b2sex+b3sportscore+εi

Next, we examined whether balancing-induced hemodynamic responses in reliable ROIs (see above) would differ between the older and younger group (equation 2). Again, the dependent variable was standardized before computation.


(2) Hemodynamics(z-transformed)i=b+0ba1gegroup+bs2ex+bs3portscore+εi

Robust linear regressions were then used to determine whether functional brain activity in the ROIs was associated with balance performance (equation 3), as indexed by the sway path of wobble board balancing (level 1). Note that this behavioral measure of balance was measured separately from the fNIRS recordings to ensure the highest possible quality of the data. Scores for balance performance and hemodynamics were standardized beforehand to yield easy to interpret standardized regression weights (*β*) that robustbase would normally not output.


(3) Performance(z-transformed)i=b+0bh1emodynamics(z-transformed)+bs2ex+bs3portscore+εi

In case that age groups differ with respect to balancing-induced hemodynamic activity in a particular ROI (equation 2), and that hemodynamic activity of this ROI does at the same time predict balance performance (equation 3), a pattern of results potentially consistent with mediation is present (James and Brett, 1984). Statistical mediation was assessed using a robust procedure proposed by Zu and Yuan (2010) implemented in R’s WRS2 v1.1-0 package (Mair and Wilcox, 2020). In essence, this procedure downweights extreme cases using a Huber-type M-estimator (Huber, 1981) in conjunction with a percentile bootstrap (*B* = 10000). Note that the Zu and Yuan (2010) method as implemented in WRS2 does not allow the inclusion of covariates, such that scores for the mediator (hemodynamics) and the criterion (balance performance) were adjusted for the covariates (biological sex and Baecke’s sport score) beforehand (residualization based on a robust linear model).

A significant regression coefficient of hemodynamics (*b*_1_) in equation 3 would suggest that a certain identified brain area is an important predictor of balance performance *regardless* of age. However, it is also possible that the effect of functional cortical activity subserving successful postural control varies by age, for instance due to a different brain reserve in older and younger participants (Li et al., 2018; Menant et al., 2020; Fettrow et al., 2021). For this reason, we calculated a moderated regression model containing an age-by-hemodynamics interaction term (equation 4).


(4) Performance(z-transformed)i=b+0bh1emodynamics

(z⁢-⁢t⁢r⁢a⁢n⁢s⁢f⁢o⁢r⁢m⁢e⁢d)+b⁢a2⁢g⁢e⁢g⁢r⁢o⁢u⁢p+b⁢a3⁢g⁢e⁢g⁢r⁢o⁢u⁢p⋅h⁢e⁢m⁢o⁢d⁢y⁢n⁢a⁢m⁢i⁢c⁢s

+bs4ex(mean-centered)+bs5portscore(mean-centered)+εi

The highest-order regression coefficient *b*_3_ in equation 4 reflects the difference in regression slopes between age groups. Interaction effects were followed up with simple slopes analysis (Dalal and Zickar, 2012). In essence, the *b*_1_ term is then the relationship between hemodynamics and balance performance when all other predictors’ values equal null. By setting the reference category of the age group to “older” (“younger”), *b*_1_ can then be interpreted as the *unique* effect of hemodynamics on balance performance exclusively for the older (younger) participants. To allow for a meaningful interpretation of the first-order effect, all predictors except of age group in equation 4 were standardized or mean centered before computing the model (Dalal and Zickar, 2012).

## Results

### Sample Characteristics

Of the initial sample, *n* = 62 participants had both complete fNIRS and behavioral data of wobble board (level 1) balancing (Table 1). After enrollment in the study, these participants also completed Baecke’s modified questionnaire of habitual physical activity (Baecke et al., 1982; Pols et al., 1995). Except for age, no significant between-group differences were detected (all *p*-values ≥ 0.09; Table 1). Although age groups did not significantly differ with respect to frequencies of biological sex and Baecke’s sport score, both variables were used as covariates of no interest in subsequent statistical models based on theory (Era et al., 2006; Daly et al., 2008; Granacher et al., 2011).

### Balance Performance in Age Groups

To gain insight into the effects of age on balance ability on the behavioral level, we next examined participants’ wobble board and Posturomed performance data. Sex and physical activity-adjusted robust linear models revealed significant group differences for all tasks (Figure 1). Note that a technical problem with the Vicon system led to missing data in the Posturomed recordings (*n* = 5 in the older group, *n* = 4 in the younger group). Likewise, not all the older participants were able to master the level 2 wobble board.

We also conducted a PCA to look whether the performance in different balance tests was driven by a latent variable. To this end, *n* = 50 (*n* = 27 older, *n* = 23 younger participants) eligible data sets with complete performance and fNIRS data were analyzed, which is a sufficient sample size provided that crucial prerequisites of PCA are met (de Winter et al., 2009; Field et al., 2012). Note that, like in the full dataset, there were no differences between age groups regarding the number of participants per group, χ^2^(1) = 0.64, *p* = 0.42, sex, χ^2^(1) = 1.03, *p* = 0.31, and Baecke’s sport score, *t*(45.33) = −0.98, *p* = 0.33.

In this *n* = 50 subset of data, the overall KMO measure (Kaiser and Rice, 1974) was 0.79 (“good” sampling adequacy), and individual KMO values ranged between 0.71 and 0.90 (“good” to “great”). Bartlett’s (1954) test of sphericity was significant, χ^2^(6) = 112.27, *p* < 0.001, suggesting sufficiently high intercorrelations among variables. Furthermore, the determinant of the correlation matrix (0.09) was greater than the necessary value of 0.00001 (Field et al., 2012), such that multicollinearity was also not a reason for concern. Finally, parallel analysis (Horn, 1965) revealed that it is appropriate to retain one factor from the data (see Table 2, for factor loadings).

Between-group differences in the extracted principal component “bipedal standing balance” were then analyzed with a robust linear model. As with wobble board and Posturomed data, there was a significant effect for age group, *β* = –1.23, 95% CI [–1.48, –0.98], *p* < 0.001, with younger adults receiving lower factor scores (reflecting better performance) than older individuals (Figure 1).

### Age-Related Differences in Cortical Activity During Balancing

**FIGURE 4:**
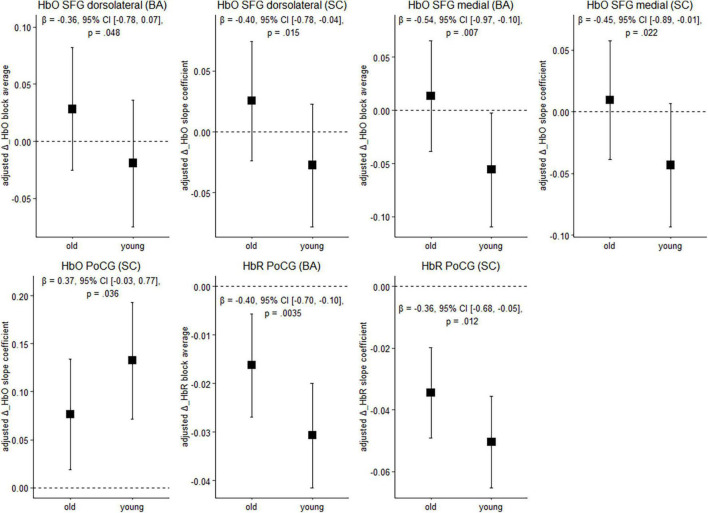
Balancing-induced neural activity in older and younger participants. Data are presented as predicted group means (corrected for the influence of sex and Baecke’s sport score) and the corresponding 95% CI (error bars). Note that the beta-coefficients reflecting the between-group differences in units of standard deviation are conceptually similar to Cohen’s *d*. Rules of thumb for interpreting this effect size are 0.2 < | *β*| < 0.5 “small,” 0.5 < | *β*| < 0.8 “medium,” and | *β*| > 0.8 “large” effects, respectively (cf. Cohen, 1988). All *p*-values that are given in the figure are not adjusted for multiple comparisons (see Supplementary Table 2, for FDR-corrected *p*-values). HbO, oxygenated hemoglobin; HbR, deoxygenated hemoglobin; BA, block average; SC, slope coefficient.

Figure 4 depicts cortical activation in a subset of ROIs during balancing on the wobble board (level 1) for both age groups. See Supplementary Table 2, for results in the remaining ROIs and FDR-corrected *p*-values.

Younger individuals exhibited lower balancing-induced hemodynamic response in the dorsolateral and medial parts of the superior frontal gyrus (SFG) compared to the older, as evidenced by smaller HbO changes. A reverse pattern was observed in the postcentral gyrus (PoCG), where cortical activity in the younger group was greater than in the older during balancing. Overall, standardized mean differences indicate that the effect of age on hemodynamics is in the small-to-medium range in the ROIs mentioned above. Of the identified regions, the effects in the medial part of the SFG (HbO, block average) and in the PoCG (HbR) are still significant after multiple comparisons correction (*p*FDR < 0.05). In the other regions mentioned above, FDR-corrected *p*-values showed a trend toward significance (*p*FDR ≤ 0.1; see Supplementary Table 2).

### The Relationship Between Hemodynamic Response and Balance Performance

Next, we focused on correlations between the balancing-induced hemodynamic response and balance performance. As outlined previously, robust linear models were calculated to unravel the association between hemodynamic changes and balance performance (see section “Materials and Methods”). In the following, we focus on regression weight *b*_1_ in equation 3, which can be interpreted as the relationship between hemodynamics and performance without considering age.

**FIGURE 5:**
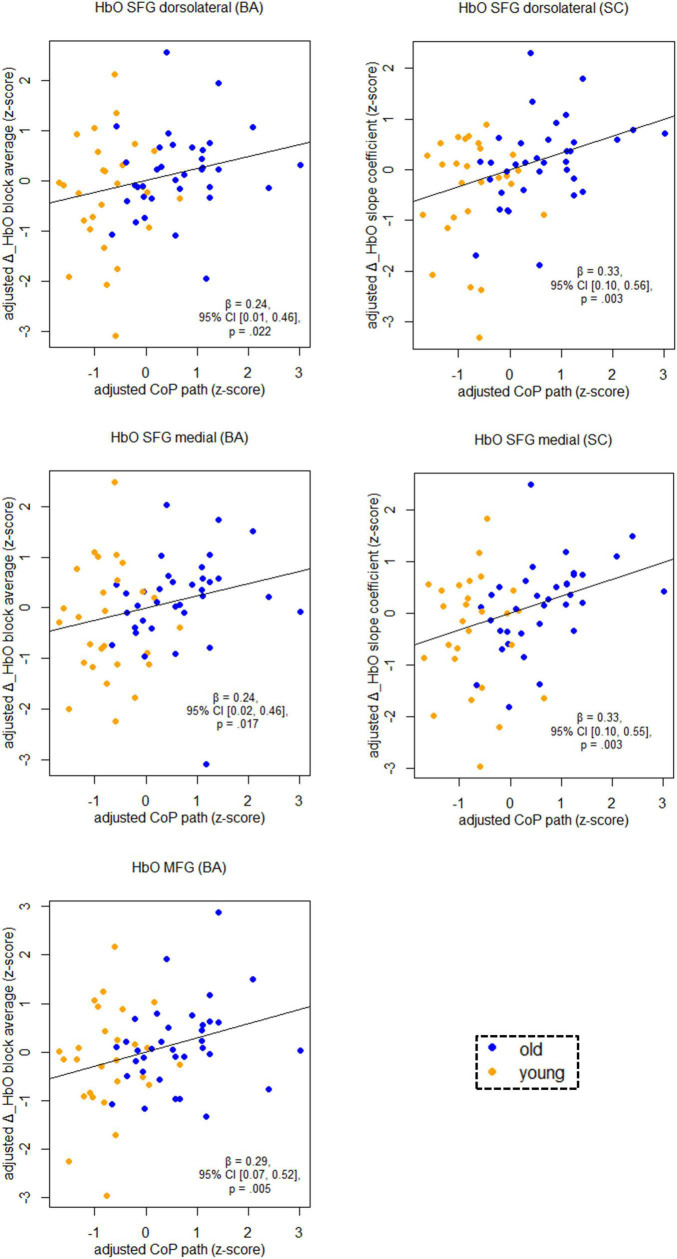
Partial regression scatterplots with lines of best fit show the relation between balancing-induced neural activity in selected ROIs and wobble board CoP path length, corrected for the influence of sex and Baecke’s sport score. Rules of thumb for interpreting the strength of associations are | *β*| < 0.30 “small,” 0.30 < | *β*| < 0.50 “medium,” and | *β*| > 0.50 “large” effects, respectively (cf. Cohen, 1988). All *p*-values that are given in the figure are not adjusted for multiple comparisons (see Supplementary Table 3, for FDR-corrected *p*-values). Abbreviations as in Figure 4.

Figure 5 shows the relationship between balancing-induced hemodynamic changes and wobble board performance (partial regression plots) in a subset of ROIs. See Supplementary Table 3, for results in all ROIs and corresponding FDR-corrected *p*-values. There were small-to-medium correlations between balance-induced changes in oxygenated hemoglobin (HbO) and wobble board performance in the SFG (dorsolateral and medial parts) and in the MFG (Figure 5). These results indicate that less activity in several prefrontal regions is associated with better balance performance across age groups. Note that the associations between the dorsolateral SFG (slope coefficient), the medial SFG (slope coefficient) and MFG (block average) did also survive correction for multiple comparisons (*p*FDR ≤ 0.03). Suggestive trends toward significance were observed for SFG dorsolateral (block average) and SFG medial (block average) (*p*FDR ≤ 0.06). No significant results were obtained in SMA, PreCG, and PoCG.

### Mediation Analysis

So far, we have demonstrated that there were two ROIs (SFG dorsolateral and medial) in which a pattern of results potentially consistent with mediation was present. Specifically, in these ROIs we found significant between-group differences regarding balance-induced hemodynamics and a significant correlation of hemodynamics and the behavioral outcome variables (James and Brett, 1984).

**FIGURE 6:**
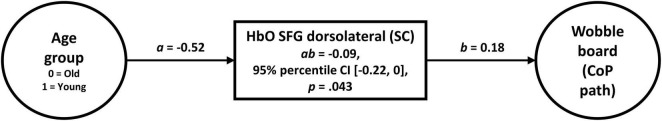
Robust mediation analysis (Zu and Yuan, 2010) revealed that balancing-induced changes in oxygenated hemoglobin (slope coefficient) in the dorsolateral part of the SFG conveys the effect of age on wobble board performance. A Huber-type weight was applied to control 5% of cases (κ = 0.05), as originally proposed by Zu and Yuan (2010). Note that the influence of sex and Baecke’s sport score was partialed out in the analysis.

To test for mediation, we conducted a robust mediation procedure as proposed by Zu and Yuan (2010) in these and the other ROIs. In the dorsolateral SFG, this analysis revealed a significant effect of changes in oxygenated hemoglobin (slope coefficient) on wobble board performance (Figure 6). This means that the younger group’s CoP path length was 0.09 standard deviations (95% percentile CI [–0.22, 0], *p* = 0.043) lower than the older group’s path length as a function of cortical activity in the dorsolateral SFG. In the other ROIs, the indirect effects were not significant (0.07 < *p* < 0.94; see Supplementary Table 5).

### The Moderating Role of Age in the Hemodynamics-Performance Relationship

Finally, we focus on the question whether age moderates the relationship between hemodynamics and balance performance. To remind the reader, simple slopes analysis in equation 4 enables us to determine the hemodynamics-performance relationship separately for each age group, and the highest-order regression term *b*_3_ represents differences in the regression slopes between age groups (Dalal and Zickar, 2012).

**FIGURE 7:**
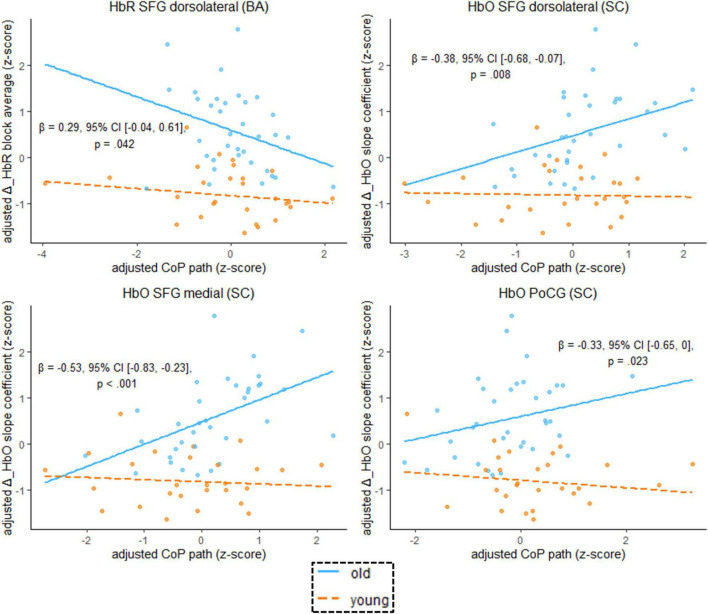
Scatterplots with lines of best fit show the relation between balancing-induced neural activity in selected ROIs and wobble board CoP path length. Compared to Figure 5, age group and an age group-by-hemodynamics interaction term were added as covariates to the linear model. The blue (orange) line of best fit can be interpreted as the unique effect of hemodynamics on balance performance exclusively for the older (younger) participants (simple slopes). Annotated regression coefficients show the between-group difference in simple slopes (i.e., the interaction effect). All *p*-values that are given in the figure are not adjusted for multiple comparisons (see Supplementary Table 4, for FDR-corrected *p*-values). Abbreviations as in Figure 4.

We found that the age-by-hemodynamics interaction term significantly predicted wobble board performance in the dorsolateral and medial parts of the SFG and in the PoCG (Figure 7). A closer look at simple main effects (Supplementary Table 4) revealed that hemodynamics was (mostly) a more important predictor of balance performance for the older than for the younger. In the older, higher hemodynamic activity in terms of increased HbO and decreased HbR was associated with worse performance in the wobble board task, as is evidenced by significant correlations (i.e., simple slopes) between hemodynamics and balance performance in the SFG (dorsolateral and medial parts), in the MFG, and in the PoCG (see Supplementary Table 4). Conversely, correlations in the younger group were mostly non-significant. With respect to deoxygenated hemoglobin (block average) in the MFG, however, simple slopes analysis revealed a meaningful hemodynamics-performance relationship both within the subsets of older (*β* = –0.33, 95% CI [–0.57, –0.09], *p* = 0.003) and younger (*β* = –0.16, 95% CI [–0.36, 0.03], *p* = 0.053) participants.

## Discussion

**FIGURE 8:**
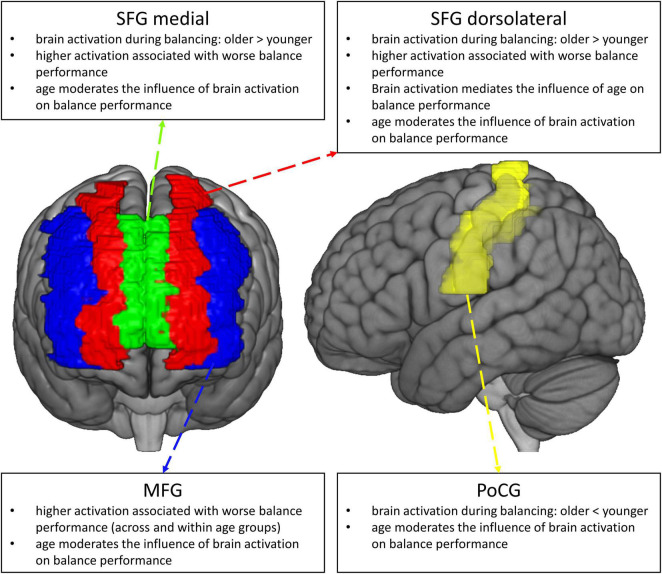
Graphical summary of the main results of the study.

Given the high prevalence and morbidity of fall accidents in older adults, it is vital to understand the neurobiological mechanisms underlying the worsening of balance control with aging. This knowledge in turn is relevant for the development of efficacious intervention strategies for the prevention of falls in the older (Seidler et al., 2010; Granacher et al., 2011; Papegaaij et al., 2014; Seals et al., 2016), for example in terms of a targeted up- or downregulation of regions being dysfunctionally activated in the aging brain (Baharlouei et al., 2020). In this paper, we used fNIRS to compare age−related changes in the hemodynamic response during balancing on an unstable device (wobble board), and we studied the behavioral implications of different patterns of functional brain activation (see Figure 8, for a graphical summary of the main results). To address these points, we employed robust statistical methods and we restricted our analyses to brain regions showing reliable hemodynamic responses during balancing (Loken and Gelman, 2017; Zuo et al., 2019; Menant et al., 2020; Yücel et al., 2021). We found that balance-induced hemodynamic activity was higher in the PFC and lower in the Postcentral Gyrus in older compared to high-functioning younger participants. Lower prefrontal neural activity was related to improved balance performance across age groups. Importantly, activity in the dorsolateral part of the SFG significantly mediated the effect of age on balance performance. Our analyses also revealed that hemodynamic activation was generally a better predictor of balance performance in the older than in the younger. Finally, MFG activity was meaningfully related to balance performance both within the older and younger subset of participants. Therefore, it might be assumed that this region is a crucial neural correlate for the control of challenging upright stance independent of age.

Our finding that older adults, compared to younger ones, showed higher activity in subregions of the PFC during balancing is in line with previous studies in posture and gait research (Lin et al., 2017; Rosso et al., 2017; Teo et al., 2018; St George et al., 2021) and consistent with predictions from influential theorical perspectives on brain activation with aging (Festini et al., 2018; Yeung and Chan, 2021). Higher activation of the PFC is commonly attributed to a decline in the aging brain’s efficiency of information processing, i.e., due to structural, functional and biochemical changes especially in (but not limited to) frontal (Wagshul et al., 2019) and classical motor coordination-related brain areas (Taubert et al., 2020) or the degradation in sensory receptors (Seidler et al., 2010; Clark, 2015; Fettrow et al., 2021). The aging-related decline in physiological function in turn leads to a reduced functional supply to cope with the demands of mastering complex movements. Against this background, it is assumed that increased PFC activation, especially in the older, reflects an attempt to recruit a higher amount of limited neural resources (Seidler et al., 2010; Fettrow et al., 2021; Yeung and Chan, 2021). A not mutually exclusive alternative to this scenario is that worsened processing of multisensory information associated with age leads to impaired automaticity of movement, thus inducing higher attentional demands (Lajoie et al., 1993; Richer and Lajoie, 2020).

Interestingly, the difference between age groups in prefrontal activation was not primarily or disproportionately driven by a hyperactivation (cf. Reuter-Lorenz and Cappell, 2008) in the older group (see Figure 4, where the 95% CI of the mean in prefrontal regions is not completely above zero). Rather, besides a slight increase of activation in the older, we observed a slight decrease of activation in the younger (Figure 4). This pattern of results resembles recent findings by St George et al. (2021) in a relatively easy bipedal task (standing on a solid surface with open eyes) under single- and, more pronounced, dual-task conditions. Therefore, in line with previous studies our findings would seem to suggest that prefrontal overactivation in the older might be a phenomenon only present in comparatively difficult balance tasks (Teo et al., 2018; St George et al., 2021) and/or when available sensory information is restricted (Lin et al., 2017).

A reversed pattern of age-group differences, namely higher activation in younger compared to older participants, was observed for the Postcentral Gyrus, a critical area for proprioception (Watson et al., 2010). To the best of our knowledge, this result has not been reported in the fNIRS literature before, probably because hemodynamic responses in PoCG were not assessed in most studies addressing aging (Rosso et al., 2017; Teo et al., 2018; Marusic et al., 2019; St George et al., 2021, but see also Lin et al., 2017). There is evidence that the higher the activation of sensorimotor-related brain areas, the higher the degree of automaticity in postural control (Herold et al., 2017b; Beretta et al., 2020). This would nicely explain why younger adults demonstrated higher activity in PoCG than older adults in the present study. In contrast to our findings, another fNIRS study by de Rond et al. (2021) has recently reported higher PoCG activation in the older compared to the younger in a game-like weight-shifting task, irrespective of task load. These differing results might, for example, be caused by the different nature of the task investigated (thus requiring different postural control strategies) or different equipment used. As we were able to obtain a highly reliable signal from the PoCG (see Supplementary Table 1), it appears unlikely that our result occurred due to unsystematic sources of error (i.e., covariation between measurement noise and actual brain activity; Vitorio et al., 2017; Wittenberg et al., 2017). Further experimental investigations are warranted to better understand the role of PoCG oxygenation in balance control.

While it is generally accepted that higher prefrontal activation during balancing compared to younger individuals reflects a robust correlate of biological aging (Seidler et al., 2010; Herold et al., 2017b; Li et al., 2018; Fettrow et al., 2021; Yeung and Chan, 2021), it is not clear whether this change in neural activation is compensatory in terms of supporting the maintenance of balance performance despite decreased (e.g., sensory, cognitive, motor) resources. Against the background of the brain-behavior-correlations presented in the present paper, it is apparent that PFC activation did not appear to benefit balance performance. Rather, we interpret prefrontal oxygenation patterns as observed in the older as ineffective compensation, reflecting a neural correlate of performance decline. In line with previous research (Teo et al., 2018), we also observed that prefrontal activity was generally a more important predictor of balance performance in the older than in the younger, indicating an age-related loading of neural resources and an increased reliance on cognitive control mechanisms.

Two findings of the present study are particularly noteworthy. To begin with, we found that balancing-induced changes in the concentration of oxygenated hemoglobin in the dorsolateral part of the SFG mediate the relationship between age and balance performance. Therefore, our data suggest that increased functional activity of the dorsolateral SFG is a mechanism that contributes to decreased postural control in aging. In line with this observation, previous studies in younger adults have shown an increased cortical activity in the SFG during simulated balance tasks in an MR scanner (Karim et al., 2014; Taube et al., 2015) and after warned and unwarned postural perturbations on a moving platform (Mihara et al., 2008). Likewise, another MRI study by Makizako et al. (2013) reported that gray matter volume in the left SFG dissociated fallers and non-fallers, thus, also substantiating the importance of this area for balance control in the older. With respect to younger adults, we have previously shown that white matter microstructure in commissural fibers connecting the hemispheres of the superior prefrontal cortex predicts learning of a challenging balance task (Lehmann et al., 2019). However, as far as we know, the findings from the present study are the first to suggest that the association between age and balance performance is mediated by SFG hemodynamics.

We also found a remarkable pattern of results for balancing-induced changes in deoxygenated hemoglobin in the MFG, a subunit within the lateral PFC. Specifically, we found that lower activation of this region was related to better balance performance in both the younger (*β* = –0.16, *p* = 0.053) and the older group (*β* = –0.33. *p* = 0.003). Therefore, these results are well in line with previous reports in indicating the importance of the MFG for balance control (Mihara et al., 2008; Karim et al., 2014). Since there was no significant difference in MFG activation between age groups (*p* = 0.18, see Supplementary Table 2), the question as to whether MFG oxygenation during balancing also reflects a mechanism involved in the aging process remains open. Although we recruited more participants than many of the existing fNIRS studies comparing younger and older adults (cf. Yeung and Chan, 2021), our study may have been under-powered to detect below-moderate effect sizes in functional activation. Evidence that the MFG might be susceptible to aging effects especially comes from studies using structural MRI. For example, Makizako et al. (2013) found that gray matter volume in the MFG was associated with the frequency of self-reported falls. Furthermore, Boisgontier et al. (2017) reported that MFG volume mediated the effects of age on standing balance as assessed with a postural disturbance task. Therefore, further work needs to be carried out to establish whether MFG oxygenation is involved in worsening of balance control with aging.

The present findings might have important implications for strategies to counteract the biological processes underlying the worsening of balance control in aging. First, our data suggest that hemodynamic responses of the brain during balancing are meaningfully related to balance performance, suggesting that fNIRS has potential as a screening tool to identify fall-prone individuals before cognitive or neurological deficits might become manifest. In this respect, activation of prefrontal brain areas might be a particularly important indicator of balance control. Second, our data could be applied to guide and tailor treatments specifically targeting the putative mechanisms underlying balance control (Seidler et al., 2010; Granacher et al., 2011; Papegaaij et al., 2014; Seals et al., 2016). For example, with respect to behavioral interventions, besides using rather specific balance or strength training (Granacher et al., 2011), our data suggest that therapeutic approaches for reducing falls by improving certain aspects of cognition (e.g., cognitive remediation) might also be promising due to their recruitment of prefrontal brain circuits (Verghese et al., 2010; Smith-Ray et al., 2015; Li et al., 2018; Sprague et al., 2021). The benefit of interventions involving a cognitive component might be even more pronounced if simultaneously combined with some form of physical training (i.e., dual-task training; see Li et al., 2018; Netz, 2019). Third, the results from our study can also be used to optimize alternative intervention approaches like non-invasive brain stimulation, where knowledge of effective stimulation sites is crucial for the efficacy of the treatment. In this regard, our findings would suggest that downregulation of PFC excitability might be particularly promising for promoting balance.

A number of potential limitations of the present study need to be considered. To begin with, we opted to use separately (and not concurrently) acquired behavioral (wobble board) data for correlational and mediation analyses. This choice was made since we used a stationary fNIRS system, and source and detector cables might therefore exert an influence on balance performance (cf. Bloem et al., 2003). Furthermore, this procedure also avoids the problem of a potential covariation between measurement noise (e.g., movement-related artifacts) and brain activity (Castermans et al., 2014; Vitorio et al., 2017; Wittenberg et al., 2017). Note, however, that CoP sway paths of wobble board balancing with and without concurrent fNIRS correlated significantly, *r* = 0.41, *p* = 0.001, thus suggesting that both balance assessments measure a similar underlying construct. A potential source of unreliability that might have influenced the results obtained is in the fNIRS method itself. Specifically, several unsystematic effects such as head motion and positioning, displacement of optodes or systemic influences (e.g., changes in heart rate and blood pressure) might have been sources of error affecting the imaging signal (Vitorio et al., 2017; Wittenberg et al., 2017; Menant et al., 2020). Although we did not directly measure these sources of noise, recent evidence suggests that signals obtained by short-distance channels share a considerable amount of variance with noise- and motion-associated parameters, therefore performing well in nuisance regression (Lühmann et al., 2020). In terms of preprocessing, it should also be noted that certain settings such as the choice of filter cut-off frequencies might have affected statistical results (Pinti et al., 2018), but this effect is expected to be not profound (Izzetoglu and Holtzer, 2020). With respect to the potential impact of data acquisition and processing, we emphasize that statistical analyses in the present paper were restricted to data from channels showing at least a moderate test-retest reliability, a basic prerequisite for generating replicable results (Loken and Gelman, 2017; Zuo et al., 2019; Menant et al., 2020). Due to the design of our study, it was unavoidable that the number of balance tests administered to participants differed between the older and the younger group; i.e., the younger performed more trials during the session. However, using non-parametric partial correlations (adjusted for age group), we found (1) no significant influence of previous Posturomed (all levels) practice on wobble board (level 1) performance (*r* = 0.04, *p* = 0.75), and no significant influence of combined Posturomed (all levels) and wobble board (all levels) practice on fNIRS results (all | *r*| ‘s ≤ 0.12, all *p*’s > 0.35; tested for SFG dorsolateral, SFG medial and PoCG). We conclude from these analyses that the amount of within-session balance practice exerted no significant influence on wobble board performance and brain activation. Another aspect to consider is that the behavioral assessments in the present study focused on standing tests with and without wobble boards, which might limit the generalizability of results. We found that the balance tasks we used in the present study heavily load on one latent factor (Table 2), but due to the complexity of the tasks it is difficult to uniquely assign this latent variable to one the different balance categories proposed by Shumway-Cook and Woollacott (2017). Notwithstanding this, there is now increasing awareness that transferability between different types of balance is small, suggesting that constructs like steady-state, proactive and reactive balance or static and dynamic balance are relatively independent from one another (Muehlbauer et al., 2012; Kümmel et al., 2016; Rizzato et al., 2021; Schedler et al., 2021). Therefore, our findings on the relationship between hemodynamics and balance performance might not be generalized to other types of balance than bipedal standing tasks. Note, however, that postural sway in standing as chosen in the present study has shown to be particularly sensitive to the influence of aging (Park et al., 2016). Unlike other studies in the area (Teo et al., 2018; St George et al., 2021), we have not examined laterality effects in brain activation during balancing (i.e., neural dedifferentiation). This was due to the fact that we aspired to generate as replicable results as possible by means of maximizing reliability of functional imaging data (Zuo et al., 2019; Menant et al., 2020). To this end, we expected that reliability would be higher when parameters are derived from larger signal averages, as suggested for instance by studies on bioelectrical signal processing (Batdorf et al., 2006). Therefore, we opted to average fNIRS signals across all channels belonging to the left and right hemisphere of the respective ROIs. Finally, the current study was not specifically designed to assess whether there would be an interaction between balancing-associated hemodynamic responses and task difficulty or the manipulation of sensory input (Lin et al., 2017; Teo et al., 2018; St George et al., 2021). Note, however, that even if the neural resources of the older have not been overloaded by our balance task, between-group differences in hemodynamics and their relatedness to behavioral outcome measures were evident.

To conclude, our study shed new light on the neural mechanisms underlying successful balance performance and how they are affected by aging. The evidence from our study suggests that neural activity – especially in prefrontal brain areas – does not only distinguish between age groups, but also constitutes an important mechanism underlying the age-related decrease in balance control. The present findings might have promising implications for healthcare professionals and coaches, as they indicate the possibility for early detection of fall-prone individuals by measuring functional brain activity during balancing. Furthermore, our work revealed promising candidate mechanisms, i.e., neural networks, that should be targeted by specifically designed physical and non-physical intervention approaches aiming to improve balance and healthy aging.

## Data Availability Statement

The raw data supporting the conclusions of this article will be made available by the authors, without undue reservation.

## Ethics Statement

The studies involving human participants were reviewed and approved by Commission cantonale d’Éthique de la Recherche sur l’être humain Vaud. The patients/participants provided their written informed consent to participate in this study.

## Author Contributions

NL: conceptualization, methodology, formal analysis, investigation, data curation, writing – original draft, and visualization. Y-AK: methodology, investigation, project administration, and writing – review and editing. MK: methodology, investigation, writing – review and editing. NA and FH: formal analysis and writing – review and editing. BD: funding acquisition and writing – review and editing. WT: funding acquisition, conceptualization, resources, methodology, supervision, and writing – review and editing. MT: funding acquisition, conceptualization, supervision, and writing – review and editing. All authors: contributed to the article and approved the submitted version.

## Conflict of Interest

The authors declare that the research was conducted in the absence of any commercial or financial relationships that could be construed as a potential conflict of interest.

## Publisher’s Note

All claims expressed in this article are solely those of the authors and do not necessarily represent those of their affiliated organizations, or those of the publisher, the editors and the reviewers. Any product that may be evaluated in this article, or claim that may be made by its manufacturer, is not guaranteed or endorsed by the publisher.

## References

[B2] Ambrose A. F. Paul G. Hausdorff J. M. (2013). Risk factors for falls among older adults: a review of the literature. *Maturitas* 75 51–61. 10.1016/j.maturitas.2013.02.009 23523272

[B3] Baecke J. A. Burema J. Frijters J. E. (1982). A short questionnaire for the measurement of habitual physical activity in epidemiological studies. *Am. J. Clin. Nutr.* 36 936–942. 10.1093/ajcn/36.5.936 7137077

[B4] Baharlouei H. Saba M. A. Shaterzadeh Yazdi M. J. Jaberzadeh S. (2020). The effect of transcranial direct current stimulation on balance in healthy young and older adults: A systematic review of the literature. *Neurophysiol. Clin.* 50 119–131. 10.1016/j.neucli.2020.01.006 32113708

[B5] Bartlett M. S. (1954). A note on the multiplying factors for various χ^2^ approximations. *J. R. Stat. Soc. Ser. B Stat. Methodol.* 16 296–298. 10.1111/j.2517-6161.1954.tb00174.x

[B6] Batdorf B. H. Feiveson A. H. Schlegel T. T. (2006). The effect of signal averaging on the reproducibility and reliability of measures of T-wave morphology. *J. Electrocardiol.* 39 266–270. 10.1016/j.jelectrocard.2005.11.004 16529767

[B7] Benjamini Y. Hochberg Y. (1995). Controlling the false discovery rate: a practical and powerful approach to multiple testing. *J. R. Stat. Soc. Ser. B Stat. Methodol.* 57 289–300. 10.1111/j.2517-6161.1995.tb02031.x

[B8] Beretta V. S. Vitório R. Nóbrega-Sousa P. Conceição N. R. Orcioli-Silva D. Pereira M. P. (2020). Effect of different intensities of transcranial direct current stimulation on postural response to external perturbation in patients with parkinson’s disease. *Neurorehabil. Neural. Repair* 34 1009–1019. 10.1177/1545968320962513 33000679

[B9] Bloem B. R. Visser J. E. Allum J. H. J. (2003). “Posturography,” in *Movement Disorders*, ed. Hallett M. (Amsterdam: Elsevier), 295–336.

[B10] Boeer J. Mueller O. Krauss I. Haupt G. Horstmann T. (2010). Reliability of a measurement technique to characterise standing properties and to quantify balance capabilities of healthy subjects on an unstable oscillatory platform (Posturomed). *Sportverletz Sportschaden* 24 40–45. 10.1055/s-0029-1245184 20229447

[B11] Boisgontier M. P. Cheval B. Chalavi S. van Ruitenbeek P. Leunissen I. Levin O. (2017). Individual differences in brainstem and basal ganglia structure predict postural control and balance loss in young and older adults. *Neurobiol. Aging* 50 47–59. 10.1016/j.neurobiolaging.2016.10.024 27875755

[B12] Brickenkamp R. Schmidt-Atzert L. Liepmann D. (2010). *Test d2 - Revision: Aufmerksamkeits- und Konzentrationstest.* Göttingen: Hogrefe.

[B13] Brigadoi S. Cooper R. J. (2015). How short is short? Optimum source-detector distance for short-separation channels in functional near-infrared spectroscopy. *Neurophotonics* 2:25005. 10.1117/1.NPh.2.2.025005 PMC4478880 26158009

[B14] Brigadoi S. Ceccherini L. Cutini S. Scarpa F. Scatturin P. Selb J. (2014). Motion artifacts in functional near-infrared spectroscopy: a comparison of motion correction techniques applied to real cognitive data. *Neuroimage* 85 181–191. 10.1016/j.neuroimage.2013.04.082 23639260 PMC3762942

[B15] Broscheid K.-C. Hamacher D. Lamprecht J. Sailer M. Schega L. (2020). Inter-session reliability of functional near-infrared spectroscopy at the prefrontal cortex while walking in multiple sclerosis. *Brain Sci.* 10:643. 10.3390/brainsci10090643 32957682 PMC7565127

[B16] Carius D. Hörnig L. Ragert P. Kaminski E. (2020). Characterizing cortical hemodynamic changes during climbing and its relation to climbing expertise. *Neurosci. Lett.* 715:134604. 10.1016/j.neulet.2019.134604 31693932

[B17] Castermans T. Duvinage M. Cheron G. Dutoit T. (2014). About the cortical origin of the low-delta and high-gamma rhythms observed in EEG signals during treadmill walking. *Neurosci. Lett.* 561 166–170. 10.1016/j.neulet.2013.12.059 24412128

[B18] Cicchetti D. V. (1994). Guidelines, criteria, and rules of thumb for evaluating normed and standardized assessment instruments in psychology. *Psychol. Assess* 6 284–290. 10.1037/1040-3590.6.4.284

[B19] Clark D. J. (2015). Automaticity of walking: functional significance, mechanisms, measurement and rehabilitation strategies. *Front. Hum. Neurosci.* 9:246. 10.3389/fnhum.2015.00246 25999838 PMC4419715

[B20] Cohen J. (1988). *Statistical power analysis for the behavioral sciences.* Hillsdale, NJ: Erlbaum.

[B21] Dalal D. K. Zickar M. J. (2012). Some common myths about centering predictor variables in moderated multiple regression and polynomial regression. *Organ. Res. Methods* 15 339–362. 10.1177/1094428111430540

[B22] Daly R. M. Ahlborg H. G. Ringsberg K. Gardsell P. Sernbo I. Karlsson M. K. (2008). Association between changes in habitual physical activity and changes in bone density, muscle strength, and functional performance in elderly men and women. *J. Am. Geriatr. Soc.* 56 2252–2260. 10.1111/j.1532-5415.2008.02039.x 19016934

[B23] de Rond V. Orcioli-Silva D. Dijkstra B. W. Orban de Xivry J.-J. Pantall A. Nieuwboer A. (2021). Compromised brain activity with age during a game-like dynamic balance task: single- vs. dual-task performance. *Front. Aging Neurosci.* 13:159. 10.3389/fnagi.2021.657308 34290599 PMC8287632

[B24] de Winter J. C. F. Dodou D. Wieringa P. A. (2009). Exploratory factor analysis with small sample sizes. *Multivar. Behav. Res.* 44 147–181. 10.1080/00273170902794206 26754265

[B25] Delbaere K. Close J. C. T. Heim J. Sachdev P. S. Brodaty H. Slavin M. J. (2010). A multifactorial approach to understanding fall risk in older people. *J. Am. Geriatr. Soc.* 58 1679–1685. 10.1111/j.1532-5415.2010.03017.x 20863327

[B26] Dijkstra B. W. Bekkers E. M. J. Gilat M. Rond V. de Hardwick R. M. Nieuwboer A. (2020). Functional neuroimaging of human postural control: a systematic review with meta-analysis. *Neurosci. Biobehav. Rev.* 115 351–362. 10.1016/j.neubiorev.2020.04.028 32407735

[B27] Era P. Sainio P. Koskinen S. Haavisto P. Vaara M. Aromaa A. (2006). Postural balance in a random sample of 7,979 subjects aged 30 years and over. *Gerontology* 52 204–213. 10.1159/000093652 16849863

[B28] Festini S. B. Zahodne L. Reuter-Lorenz P. A. (2018). “Theoretical Perspectives on Age Differences in Brain Activation: HAROLD, PASA, CRUNCH—How Do They STAC Up?” in *Oxford Research Encyclopedia of Psychology*, ed. Braddick O. (Oxford: Oxford University Press).

[B29] Fettrow T. Hupfeld K. Tays G. Clark D. J. Reuter-Lorenz P. A. Seidler R. D. (2021). Brain activity during walking in older adults: Implications for compensatory versus dysfunctional accounts. *Neurobiol. Aging* 105 349–364. 10.1016/j.neurobiolaging.2021.05.015 34182403 PMC8338893

[B30] Field A. P. Wilcox R. R. (2017). Robust statistical methods: a primer for clinical psychology and experimental psychopathology researchers. *Behav. Res. Ther.* 98 19–38. 10.1016/j.brat.2017.05.013 28577757

[B31] Field A. Miles J. Field Z. (2012). *Discovering statistics using R.* Los Angeles, CA: Sage.

[B32] Fleiss J. L. (1986). *The Design and Analysis of Clinical Experiments.* New York, NY: Wiley.

[B33] Florence C. S. Bergen G. Atherly A. Burns E. Stevens J. Drake C. (2018). Medical costs of fatal and nonfatal falls in older adults. *J. Am. Geriatr. Soc.* 66 693–698. 10.1111/jgs.15304 29512120 PMC6089380

[B34] Fujita H. Kasubuchi K. Wakata S. Hiyamizu M. Morioka S. (2016). Role of the frontal cortex in standing postural sway tasks while dual-tasking: a functional near-infrared spectroscopy study examining working memory capacity. *Biomed. Res. Int.* 2016:7053867. 10.1155/2016/7053867 27034947 PMC4791508

[B35] Gagnon L. Perdue K. Greve D. N. Goldenholz D. Kaskhedikar G. Boas D. A. (2011). Improved recovery of the hemodynamic response in diffuse optical imaging using short optode separations and state-space modeling. *Neuroimage* 56 1362–1371. 10.1016/j.neuroimage.2011.03.001 21385616 PMC3085546

[B36] Gevins A. Cutillo B. (1993). Spatiotemporal dynamics of component processes in human working memory. *Electroencephalogr. Clin. Neurophysiol.* 87 128–143. 10.1016/0013-4694(93)90119-G 7691540

[B37] Granacher U. Muehlbauer T. Gollhofer A. Kressig R. W. Zahner L. (2011). An intergenerational approach in the promotion of balance and strength for fall prevention - a mini-review. *Gerontology* 57 304–315. 10.1159/000320250 20720401

[B38] Herold F. Aye N. Hamacher D. Schega L. (2019). Towards the neuromotor control processes of steady-state and speed-matched treadmill and overground walking. *Brain Topogr.* 32 472–476. 10.1007/s10548-019-00699-8 30680671

[B39] Herold F. Orlowski K. Börmel S. Müller N. G. (2017a). Cortical activation during balancing on a balance board. *Hum. Mov. Sci.* 51 51–58. 10.1016/j.humov.2016.11.002 27846398

[B40] Herold F. Wiegel P. Scholkmann F. Müller N. G. (2018). Applications of functional near-infrared spectroscopy (fNIRS) neuroimaging in exercise-cognition science: a systematic, methodology-focused review. *J. Clin. Med.* 7:466. 10.3390/jcm7120466 30469482 PMC6306799

[B41] Herold F. Wiegel P. Scholkmann F. Thiers A. Hamacher D. Schega L. (2017b). Functional near-infrared spectroscopy in movement science: a systematic review on cortical activity in postural and walking tasks. *Neurophotonics* 4:41403. 10.1117/1.NPh.4.4.041403 PMC5538329 28924563

[B42] Horn J. L. (1965). A rationale and test for the number of factors in factor analysis. *Psychometrika* 30 179–185. 10.1007/BF02289447 14306381

[B43] Huber P. J. (1981). *Robust statistics.* New York, NY: Wiley.

[B44] Hunter S. K. Pereira H. M. Keenan K. G. (2016). The aging neuromuscular system and motor performance. *J. Appl. Physiol.* 121 982–995. 10.1152/japplphysiol.00475.2016 27516536 PMC5142309

[B45] Huppert T. J. Diamond S. G. Franceschini M. A. Boas D. A. (2009). HomER: a review of time-series analysis methods for near-infrared spectroscopy of the brain. *Appl. Opt.* 48 D280–D298. 10.1364/AO.48.00D280 19340120 PMC2761652

[B46] Hwang H.-J. Choi H. Kim J.-Y. Chang W.-D. Kim D.-W. Kim K. (2016). Toward more intuitive brain-computer interfacing: classification of binary covert intentions using functional near-infrared spectroscopy. *J. Biomed. Opt.* 21:91303. 10.1117/1.JBO.21.9.091303 27050535

[B47] Izzetoglu M. Holtzer R. (2020). Effects of processing methods on fNIRS signals assessed during active walking tasks in older adults. *IEEE Trans. Neural. Syst. Rehabil. Eng.* 28 699–709. 10.1109/TNSRE.2020.2970407 32070987 PMC7768789

[B48] James L. R. Brett J. M. (1984). Mediators, moderators, and tests for mediation. *J. Appl. Psychol.* 69 307–321. 10.1037/0021-9010.69.2.307

[B49] Kaiser H. F. Rice J. (1974). Little Jiffy, Mark IV. *Educ. Psychol. Meas.* 34 111–117. 10.1177/001316447403400115

[B50] Kannus P. Parkkari J. Koskinen S. Niemi S. Palvanen M. Järvinen M. (1999). Fall-induced injuries and deaths among older adults. *JAMA* 281 1895–1899. 10.1001/jama.281.20.1895 10349892

[B51] Kapteyn T. S. Bles W. Njiokiktjien C. J. Kodde L. Massen C. H. Mol J. M. (1983). Standardization in platform stabilometry being a part of posturography. *Agressologie* 24 321–326. 6638321

[B52] Karim H. T. Sparto P. J. Aizenstein H. J. Furman J. M. Huppert T. J. Erickson K. I. (2014). Functional MR imaging of a simulated balance task. *Brain Res.* 1555 20–27. 10.1016/j.brainres.2014.01.033 24480476 PMC4001860

[B53] Karim H. Schmidt B. Dart D. Beluk N. Huppert T. (2012). Functional near-infrared spectroscopy (fNIRS) of brain function during active balancing using a video game system. *Gait. Posture* 35 367–372. 10.1016/j.gaitpost.2011.10.007 22078300 PMC3294084

[B54] Koller M. Stahel W. A. (2017). Nonsingular subsampling for regression S estimators with categorical predictors. *Comput. Stat.* 32 631–646. 10.1007/s00180-016-0679-x

[B55] Kümmel J. Kramer A. Giboin L.-S. Gruber M. (2016). Specificity of balance training in healthy individuals: a systematic review and meta-analysis. *Sports Med.* 46 1261–1271. 10.1007/s40279-016-0515-z 26993132

[B56] Kurz M. J. Wilson T. W. Arpin D. J. (2012). Stride-time variability and sensorimotor cortical activation during walking. *Neuroimage* 59 1602–1607. 10.1016/j.neuroimage.2011.08.084 21920441

[B57] Lajoie Y. Teasdale N. Bard C. Fleury M. (1993). Attentional demands for static and dynamic equilibrium. *Exp. Brain Res.* 97 139–144. 10.1007/BF00228824 8131825

[B58] Lehmann N. Tolentino-Castro J. W. Kaminski E. Ragert P. Villringer A. Taubert M. (2019). Interindividual differences in gray and white matter properties are associated with early complex motor skill acquisition. *Hum. Brain Mapp.* 40 4316–4330. 10.1002/hbm.24704 31264300 PMC6865641

[B59] Li K. Z. H. Bherer L. Mirelman A. Maidan I. Hausdorff J. M. (2018). Cognitive involvement in balance, gait and dual-tasking in aging: a focused review from a neuroscience of aging perspective. *Front. Neurol.* 9:913. 10.3389/fneur.2018.00913 30425679 PMC6219267

[B60] Lin C.-C. Barker J. W. Sparto P. J. Furman J. M. Huppert T. J. (2017). Functional near-infrared spectroscopy (fNIRS) brain imaging of multi-sensory integration during computerized dynamic posturography in middle-aged and older adults. *Exp. Brain Res.* 235 1247–1256. 10.1007/s00221-017-4893-8 28197672 PMC5494712

[B61] Loken E. Gelman A. (2017). Measurement error and the replication crisis. *Science* 355 584–585. 10.1126/science.aal3618 28183939

[B62] Lühmann A. von Li X. Müller K.-R. Boas D. A. Yücel M. A. (2020). Improved physiological noise regression in fNIRS: a multimodal extension of the general linear model using temporally embedded canonical correlation analysis. *Neuroimage* 208:116472. 10.1016/j.neuroimage.2019.116472 31870944 PMC7703677

[B63] Maechler M. Rousseeuw P. Croux C. Todorov V. Ruckstuhl A. Salibian-Barrera M. (2020). *robustbase: Basic Robust Statistics. R package version 0.93-6.*

[B64] Mair P. Wilcox R. (2020). Robust statistical methods in R using the WRS2 package. *Behav. Res. Methods* 52 464–488. 10.3758/s13428-019-01246-w 31152384

[B65] Makizako H. Shimada H. Doi T. Park H. Yoshida D. Uemura K. (2013). Poor balance and lower gray matter volume predict falls in older adults with mild cognitive impairment. *BMC Neurol.* 13:3. 10.1186/1471-2377-13-102 23915144 PMC3750260

[B66] Mandrick K. Derosiere G. Dray G. Coulon D. Micallef J.-P. Perrey S. (2013). Utilizing slope method as an alternative data analysis for functional near-infrared spectroscopy-derived cerebral hemodynamic responses. *Int. J. Ind. Ergon.* 43 335–341. 10.1016/j.ergon.2013.05.003

[B67] Maronna R. A. Martin D. Yohai V. J. (2006). *Robust statistics: Theory and methods.* Chichester: Wiley.

[B68] Marusic U. Taube W. Morrison S. A. Biasutti L. Grassi B. Pauw K. (2019). Aging effects on prefrontal cortex oxygenation in a posture-cognition dual-task: an fNIRS pilot study. *Eur. Rev. Aging Phys. Act* 16:2. 10.1186/s11556-018-0209-7 30655911 PMC6329111

[B69] McGraw K. O. Wong S. P. (1996). Forming inferences about some intraclass correlation coefficients. *Psychol. Methods* 1 30–46. 10.1037/1082-989X.1.1.30

[B70] Menant J. C. Maidan I. Alcock L. Al-Yahya E. Cerasa A. Clark D. J. (2020). A consensus guide to using functional near-infrared spectroscopy in posture and gait research. *Gait. Posture* 82 254–265. 10.1016/j.gaitpost.2020.09.012 32987345

[B71] Mihara M. Miyai I. Hatakenaka M. Kubota K. Sakoda S. (2008). Role of the prefrontal cortex in human balance control. *Neuroimage* 43 329–336. 10.1016/j.neuroimage.2008.07.029 18718542

[B72] Molavi B. Dumont G. A. (2012). Wavelet-based motion artifact removal for functional near-infrared spectroscopy. *Physiol. Meas.* 33 259–270. 10.1088/0967-3334/33/2/259 22273765

[B73] Muehlbauer T. Besemer C. Wehrle A. Gollhofer A. Granacher U. (2012). Relationship between strength, power and balance performance in seniors. *Gerontology* 58 504–512. 10.1159/000341614 22922168

[B74] Netz Y. (2019). Is there a preferred mode of exercise for cognition enhancement in older age-a narrative review. *Front. Med.* 6:57. 10.3389/fmed.2019.00057 30984760 PMC6450219

[B75] Oostenveld R. Praamstra P. (2001). The five percent electrode system for high-resolution EEG and ERP measurements. *Clin. Neurophysiol.* 112 713–719. 10.1016/S1388-2457(00)00527-7 11275545

[B76] Papegaaij S. Taube W. Baudry S. Otten E. Hortobágyi T. (2014). Aging causes a reorganization of cortical and spinal control of posture. *Front. Aging Neurosci.* 6:28. 10.3389/fnagi.2014.00028 24624082 PMC3939445

[B77] Park J.-H. Mancini M. Carlson-Kuhta P. Nutt J. G. Horak F. B. (2016). Quantifying effects of age on balance and gait with inertial sensors in community-dwelling healthy adults. *Exp. Gerontol.* 85 48–58. 10.1016/j.exger.2016.09.018 27666186 PMC5101181

[B78] Perrey S. Besson P. (2018). “Studying brain activity in sports performance: Contributions and issues,” in *Sport and the Brain: The Science of Preparing, Enduring and Winning : Part C*, eds Marcora S. Sarkar M. (Cambridge, MA: Academic Press), 247–267. 10.1016/bs.pbr.2018.07.004 30390834

[B79] Pinti P. Scholkmann F. Hamilton A. Burgess P. Tachtsidis I. (2018). Current status and issues regarding pre-processing of fNIRS neuroimaging data: an investigation of diverse signal filtering methods within a general linear model framework. *Front. Hum. Neurosci.* 12:505. 10.3389/fnhum.2018.00505 30687038 PMC6336925

[B80] Poline J.-B. Brett M. (2012). The general linear model and fMRI: does love last forever? *Neuroimage* 62 871–880. 10.1016/j.neuroimage.2012.01.133 22343127

[B81] Pols M. A. Peeters P. H. Bueno-De-Mesquita H. B. Ocké M. C. Wentink C. A. Kemper H. C. (1995). Validity and repeatability of a modified Baecke questionnaire on physical activity. *Int. J. Epidemiol.* 24 381–388. 10.1093/ije/24.2.381 7635600

[B82] R Development Core Team (2018). *R. A language and environment for statistical computing: A language and environment for statistical computing.* Vienna: R Foundation for Statistical Computing.

[B83] Reuter-Lorenz P. A. Cappell K. A. (2008). Neurocognitive aging and the compensation hypothesis. *Curr. Dir. Psychol. Sci.* 17 177–182. 10.1111/j.1467-8721.2008.00570.x

[B84] Richer N. Lajoie Y. (2020). Automaticity of postural control while dual-tasking revealed in young and older adults. *Exp. Aging Res.* 46 1–21. 10.1080/0361073X.2019.1693044 31744403

[B85] Rizzato A. Paoli A. Andretta M. Vidorin F. Marcolin G. (2021). Are static and dynamic postural balance assessments two sides of the same coin? a cross-sectional study in the older adults. *Front. Physiol.* 12:438. 10.3389/fphys.2021.681370 34267673 PMC8277194

[B86] Rolls E. T. Joliot M. Tzourio-Mazoyer N. (2015). Implementation of a new parcellation of the orbitofrontal cortex in the automated anatomical labeling atlas. *Neuroimage* 122 1–5. 10.1016/j.neuroimage.2015.07.075 26241684

[B87] Rosso A. L. Cenciarini M. Sparto P. J. Loughlin P. J. Furman J. M. Huppert T. J. (2017). Neuroimaging of an attention demanding dual-task during dynamic postural control. *Gait. Posture* 57 193–198. 10.1016/j.gaitpost.2017.06.013 28662465 PMC5585862

[B88] Salibián-Barrera M. van Aelst S. Willems G. (2008). Fast and robust bootstrap. *Stat. Methods Appt.* 17 41–71. 10.1007/s10260-007-0048-6

[B89] Schedler S. Abeck E. Muehlbauer T. (2021). Relationships between types of balance performance in healthy individuals: role of age. *Gait. Posture* 84 352–356. 10.1016/j.gaitpost.2021.01.008 33465735

[B90] Schellig D. Schächtele B. (2009). *Visueller und verbaler Merkfähigkeitstest (VVM).* Frankfurt: Pearson.

[B91] Schmitt T. A. (2011). Current methodological considerations in exploratory and confirmatory factor analysis. *J. Psychoeduc. Assess* 29 304–321. 10.1177/0734282911406653

[B92] Scholkmann F. Wolf M. (2013). General equation for the differential pathlength factor of the frontal human head depending on wavelength and age. *J. Biomed. Opt.* 18:105004. 10.1117/1.JBO.18.10.105004 24121731

[B93] Seals D. R. Justice J. N. LaRocca T. J. (2016). Physiological geroscience: targeting function to increase healthspan and achieve optimal longevity. *J. Physiol.* 594 2001–2024. 10.1113/jphysiol.2014.282665 25639909 PMC4933122

[B94] Seidel O. Carius D. Roediger J. Rumpf S. Ragert P. (2019). Changes in neurovascular coupling during cycling exercise measured by multi-distance fNIRS: a comparison between endurance athletes and physically active controls. *Exp. Brain Res.* 237 2957–2972. 10.1007/s00221-019-05646-4 31506708 PMC6794243

[B95] Seidler R. D. Bernard J. A. Burutolu T. B. Fling B. W. Gordon M. T. Gwin J. T. (2010). Motor control and aging: links to age-related brain structural, functional, and biochemical effects. *Neurosci. Biobehav. Rev.* 34 721–733. 10.1016/j.neubiorev.2009.10.005 19850077 PMC2838968

[B96] Shumway-Cook A. Woollacott M. H. (2017). *Motor control: Translating research into clinical practice.* Philadelphia, PA: Wolters Kluwer.

[B97] Smith-Ray R. L. Hughes S. L. Prohaska T. R. Little D. M. Jurivich D. A. Hedeker D. (2015). Impact of cognitive training on balance and gait in older adults. *J. Gerontol. B Psychol. Sci. Soc. Sci.* 70 357–366. 10.1093/geronb/gbt097 24192586 PMC4542642

[B98] Sprague B. N. Phillips C. B. Ross L. A. (2021). Cognitive training attenuates decline in physical function across 10 Years. *J. Gerontol. B Psychol. Sci. Soc. Sci.* 76 1114–1124. 10.1093/geronb/gbaa072 32484891 PMC8496695

[B99] St George R. J. Hinder M. R. Puri R. Walker E. Callisaya M. L. (2021). Functional near-infrared spectroscopy reveals the compensatory potential of pre-frontal cortical activity for standing balance in young and older adults. *Neuroscience* 452 208–218. 10.1016/j.neuroscience.2020.10.027 33197501

[B100] Strangman G. E. Li Z. Zhang Q. (2013). Depth sensitivity and source-detector separations for near infrared spectroscopy based on the Colin27 brain template. *PLoS One* 8:e66319. 10.1371/journal.pone.0066319 23936292 PMC3731322

[B101] Stuart S. Belluscio V. Quinn J. F. Mancini M. (2019). Pre-frontal cortical activity during walking and turning is reliable and differentiates across young, older adults and people with parkinson’s disease. *Front. Neurol.* 10:536. 10.3389/fneur.2019.00536 31191434 PMC6540937

[B102] Tachtsidis I. Scholkmann F. (2016). False positives and false negatives in functional near-infrared spectroscopy: issues, challenges, and the way forward. *Neurophotonics* 3:31405. 10.1117/1.NPh.3.3.031405 PMC4791590 27054143

[B103] Takakura H. Nishijo H. Ishikawa A. Shojaku H. (2015). Cerebral hemodynamic responses during dynamic posturography: analysis with a multichannel near-infrared spectroscopy system. *Front. Hum. Neurosci.* 9:620. 10.3389/fnhum.2015.00620 26635574 PMC4647449

[B104] Takakusaki K. (2017). Functional neuroanatomy for posture and gait control. *J. Mov. Disord.* 10 1–17. 10.14802/jmd.16062 28122432 PMC5288669

[B105] Taube W. Leukel C. Gollhofer A. (2008). Influence of enhanced visual feedback on postural control and spinal reflex modulation during stance. *Exp. Brain Res.* 188 353–361. 10.1007/s00221-008-1370-4 18421451

[B106] Taube W. Mouthon M. Leukel C. Hoogewoud H.-M. Annoni J.-M. Keller M. (2015). Brain activity during observation and motor imagery of different balance tasks: an fMRI study. *Cortex* 64 102–114. 10.1016/j.cortex.2014.09.022 25461711

[B107] Taubert M. Roggenhofer E. Melie-Garcia L. Muller S. Lehmann N. Preisig M. (2020). Converging patterns of aging-associated brain volume loss and tissue microstructure differences. *Neurobiol. Aging* 88 108–118. 10.1016/j.neurobiolaging.2020.01.006 32035845

[B108] Teo W.-P. Goodwill A. M. Hendy A. M. Muthalib M. Macpherson H. (2018). Sensory manipulation results in increased dorsolateral prefrontal cortex activation during static postural balance in sedentary older adults: An fNIRS study. *Brain Behav.* 8:e01109. 10.1002/brb3.1109 30230687 PMC6192391

[B109] Tinetti M. E. Williams C. S. (1998). The effect of falls and fall injuries on functioning in community-dwelling older persons. *J. Gerontol. A Biol. Sci. Med. Sci.* 53 M112–M119. 10.1093/gerona/53a.2.m112 9520917

[B110] Tzourio-Mazoyer N. Landeau B. Papathanassiou D. Crivello F. Etard O. Delcroix N. (2002). Automated anatomical labeling of activations in SPM using a macroscopic anatomical parcellation of the MNI MRI single-subject brain. *Neuroimage* 15 273–289. 10.1006/nimg.2001.0978 11771995

[B111] Verghese J. Mahoney J. Ambrose A. F. Wang C. Holtzer R. (2010). Effect of cognitive remediation on gait in sedentary seniors. *J. Gerontol. A Biol. Sci. Med. Sci.* 65 1338–1343. 10.1093/gerona/glq127 20643703

[B112] Villringer A. Chance B. (1997). Non-invasive optical spectroscopy and imaging of human brain function. *Trends Neurosci.* 20 435–442. 10.1016/S0166-2236(97)01132-6 9347608

[B113] Vitorio R. Stuart S. Rochester L. Alcock L. Pantall A. (2017). fNIRS response during walking - Artefact or cortical activity? A systematic review. *Neurosci. Biobehav. Rev.* 83 160–172. 10.1016/j.neubiorev.2017.10.002 29017917

[B114] Wagshul M. E. Lucas M. Ye K. Izzetoglu M. Holtzer R. (2019). Multi-modal neuroimaging of dual-task walking: Structural MRI and fNIRS analysis reveals prefrontal grey matter volume moderation of brain activation in older adults. *Neuroimage* 189 745–754. 10.1016/j.neuroimage.2019.01.045 30710680 PMC6422701

[B115] Wälchli M. Ruffieux J. Mouthon A. Keller M. Taube W. (2018). Is young age a limiting factor when training balance? effects of child-oriented balance training in children and adolescents. *Pediatr. Exerc. Sci.* 30 176–184. 10.1123/pes.2017-0061 28605259

[B116] Watson C. Paxinos G. Kirkcaldie M. (2010). *The brain: An introduction to functional neuroanatomy.* Amsterdam: Elsevier.

[B117] Willems R. M. W. Cristia A. (2018). “Hemodynamic Methods: fMRI and fNIRS,” in *Research methods in psycholinguistics and the neurobiology of language: A practical guide*, eds de Groot A. M. B. Hagoort P. (Hoboken, NJ: Wiley Blackwell), 266–287.

[B118] Wittenberg E. Thompson J. Nam C. S. Franz J. R. (2017). Neuroimaging of human balance control: a systematic review. *Front. Hum. Neurosci.* 11:170. 10.3389/fnhum.2017.00170 28443007 PMC5385364

[B119] Ye J. C. Tak S. Jang K. E. Jung J. Jang J. (2009). NIRS-SPM: statistical parametric mapping for near-infrared spectroscopy. *Neuroimage* 44 428–447. 10.1016/j.neuroimage.2008.08.036 18848897

[B120] Yeung M. K. Chan A. S. (2021). A systematic review of the application of functional near-infrared spectroscopy to the study of cerebral hemodynamics in healthy aging. *Neuropsychol. Rev.* 31 139–166. 10.1007/s11065-020-09455-3 32959167

[B121] Yohai V. J. (1987). High breakdown-point and high efficiency robust estimates for regression. *Ann. Statist.* 15 642–656. 10.1214/aos/1176350366

[B122] Yücel M. A. Lühmann A. V. Scholkmann F. Gervain J. Dan I. Ayaz H. (2021). Best practices for fNIRS publications. *Neurophotonics* 8:12101. 10.1117/1.NPh.8.1.012101 PMC7793571 33442557

[B123] Zimeo Morais G. A. Balardin J. B. Sato J. R. (2018). fNIRS Optodes’ Location Decider (fOLD): a toolbox for probe arrangement guided by brain regions-of-interest. *Sci. Rep.* 8:3341. 10.1038/s41598-018-21716-z 29463928 PMC5820343

[B124] Zu J. Yuan K.-H. (2010). Local influence and robust procedures for mediation analysis. *Multivar. Behav. Res.* 45 1–44. 10.1080/00273170903504695 26789083

[B125] Zuo X.-N. Xu T. Milham M. P. (2019). Harnessing reliability for neuroscience research. *Nat. Hum. Behav.* 3 768–771. 10.1038/s41562-019-0655-x 31253883

