# Supplementary material for: Brain Activation During Active Balancing and Its Behavioral Relevance in Younger and Older Adults: A Functional Near-Infrared Spectroscopy (fNIRS) Study
Source: Front Aging Neurosci. 2022 Mar 25;14:828474. doi: 10.3389/fnagi.2022.828474 (PMC8997341; doi:10.3389/fnagi.2022.828474)
Supplement: Supplementary file 1 [file Data_Sheet_1.PDF]

Supplementary Material

Test-retest reliability of balancing-induced hemodynamic responses

Supplementary Table 1. The figure legends are required to have the same font as the main text, 12 point normal Times New Roman, single spaced. Please use a single paragraph for each legend and prepare the figures keeping in mind the PDF layout. Test-retest reliability of balancing-induced hemodynamic responses. All channels belonging to a particular aal2 atlas ROI were first averaged. ROI-wise reliability was then assessed using the two-way mixed model ICC for average measurements, where agreement was defined in terms of consistency (McGraw and Wong, 1996). In line with ICC cut-off values proposed in the literature (Fleiss, 1986; Cicchetti, 1994), we excluded ROIs with an ICC ≤ 0.4 from further statistical analyses. Note that negative ICC values are reported as .00 (Baldwin et al., 2005).

HbO = oxygenated hemoglobin, HbR = deoxygenated hemoglobin, BA = block average, SC = slope coefficient

Abbreviations of ROIs as in main manuscript, Table 1.

ROI	ICC
PreCG_HbO_BA	ICC(A,2) = 0.33, *p* = .13
PreCG_HbO_SC	ICC(A,2) = 0.38, *p* = .09
PreCG_HbR_BA	ICC(A,2) = 0.44, *p* = .05
PreCG_HbR_SC	ICC(A,2) = 0.77, *p* < .001
SFG_HbO_BA	ICC(A,2) = 0.46, *p* = .03
SFG_HbO_SC	ICC(A,2) = 0.40, *p* = .06
SFG_HbR_BA	ICC(A,2) = 0.43, *p* = .055
SFG_HbR_SC	ICC(A,2) = 0.23, *p* = .23
MFG_HbO_BA	ICC(A,2) = 0.40, *p* = .06
MFG_HbO_BA	ICC(A,2) = 0.19, *p* = .26
MFG_HbR_BA	ICC(A,2) = 0.57, *p* = .009
MFG_HbR_BA	ICC(A,2) = 0.21, *p* = .25
SMA_HbO_BA	ICC(A,2) = 0.31, *p* = .14
SMA_HbO_SC	ICC(A,2) = 0.30, *p* = .15
SMA_HbR_BA	ICC(A,2) = 0.62, *p* = .004
SMA_HbR_SC	ICC(A,2) = 0.77, *p* < .001
SFGmedial_HbO_BA	ICC(A,2) = 0.42, *p* = .05
SFGmedial_HbO_SC	ICC(A,2) = 0.41, *p* = .06
SFGmedial_HbR_BA	ICC(A,2) = 0.25, *p* = .20
SFGmedial_HbR_SC	ICC(A,2) = 0.09, *p* = .40
PoCG_HbO_BA	ICC(A,2) = 0.22, *p* = .24
PoCG_HbO_SC	ICC(A,2) = 0.48, *p* = .03
PoCG_HbR_BA	ICC(A,2) = 0.52, *p* = .02
PoCG_HbR_SC	ICC(A,2) = 0.81, *p* < .001
MTG_L_HbO_BA	ICC(A,2) = 0, *p* = .90
MTG_L_HbO_BA	ICC(A,2) = 0, *p* = .83
MTG_L_HbR_BA	ICC(A,2) = 0, *p* = .73
MTG_L_HbR_SC	ICC(A,2) = 0, *p* = .61

Age-related differences in cortical activity during balancing

Supplementary Table 2. Robust regression analysis (Yohai, 1987; Koller and Stahel, 2017) of age group (old vs. young) as predictor of balancing-induced hemodynamics in six ROIs of the aal2 atlas, corrected for the influence of sex and Baecke's sport score. Standard errors (and associated test statistics and *p*-values) and confidence intervals were estimated based on *B* = 10000 bootstrap replications (Salibián-Barrera et al., 2008). Note that the regression coefficient of age group corresponds to the between-group difference in units of pooled standard deviation and is thus conceptually similar to the effect size *d*. Rules of thumb for interpreting this effect size are $0.2 < |\beta| < 0.5$ ''small'', $0.5 < |\beta| < 0.8$ ''medium'', and $|\beta| > 0.8$ ''large'' effects, respectively (cf. Cohen, 1988). Multiple testing correction based on the false discovery rate (FDR) was applied according to the procedure proposed by Benjamini and Hochberg (1995). Abbreviations as in Supplementary Table 1.

ROI	β (95% CI)	SE	*p* (unc.)	*p* (FDR-corr.)
PreCG_HbR_BA	-0.27 [-0.64, 0.08]	0.18	.06	.10
PreCG_HbR_SC	-0.20 [-0.49, 0.09]	0.15	.09	.12
SFG_HbO_BA	-0.36 [-0.78, 0.07]	0.21	.05	.10
SFG_HbO_SC	-0.40 [-0.78, -0.04]	0.19	.02	.052
SFG_HbR_BA	0.11 [-0.26, 0.48]	0.19	.29	.29
MFG_HbO_BA	-0.23 [-0.64, 0.19]	0.21	.14	.16
MFG_HbR_BA	-0.24 [-0.71, 0.25]	0.24	.16	.18
SMA_HbR_BA	0.27 [-0.12, 0.66]	0.20	.09	.12
SMA_HbR_SC	0.33 [-0.08, 0.74]	0.21	.06	.10
SFGmedial_HbO_BA	-0.54 [-0.97, -0.10]	0.22	.007	.04
SFGmedial_HbO_SC	-0.45 [-0.89, -0.01]	0.22	.02	.052
PoCG_HbO_SC	0.37 [-0.03, 0.77]	0.20	.04	.08
PoCG_HbR_BA	-0.40 [-0.70, -0.10]	0.15	.004	.04
PoCG_HbR_SC	-0.36 [-0.68, -0.05]	0.16	.01	.04

Relationship between hemodynamics and balance performance

Supplementary Table 3. Robust regression analysis (Yohai, 1987; Koller and Stahel, 2017) of balancing-induced hemodynamics as predictor of wobble board performance (CoP path length), corrected for the influence of sex and Baecke's sport score. EMM refers to the covariate-adjusted means of each age group (as in Figure 3, main manuscript). Standard errors (and associated test statistics and *p*-values) and confidence intervals were estimated based on B = 10000 bootstrap replications (Salibián-Barrera et al., 2008). Rules of thumb for interpreting the strength of associations are $|\beta| < 0.30$ ‘‘small”, $0.30 < |\beta| < 0.50$ ‘‘medium”, and $|\beta| > 0.50$ ‘‘large” effects, respectively (cf. Cohen, 1988). Multiple testing correction based on the false discovery rate (FDR) was applied according to the procedure proposed by Benjamini and Hochberg (1995). Abbreviations as in Supplementary Table 1.

ROI	EMM_{old} (95% CI)	EMM_{young} (95% CI)	β (95% CI)	SE	*p* (unc.)	*p* (FDR-corr.)
PreCG_HbR_BA	-0.01 [-0.02, 0.00]	-0.02 [-0.03, -0.01]	0.002 [-0.24, 0.24]	0.12	.50	.50
PreCG_HbR_SC	-0.02 [-0.04, -0.01]	-0.03 [-0.05, -0.02]	0.07 [-0.18, 0.31]	0.13	.29	.36
SFG_HbO_BA	0.03 [-0.03, 0.08]	-0.02 [-0.07, 0.04]	0.24 [0.01, 0.46]	0.12	.02	.06
SFG_HbO_SC	0.03 [-0.02, 0.07]	-0.03 [-0.08, 0.02]	0.33 [0.10, 0.56]	0.12	.003	.02
SFG_HbR_BA	-0.02 [-0.04, -0.01]	-0.02 [-0.03, 0.00]	-0.12 [-0.36, 0.11]	0.12	.16	.31
MFG_HbO_BA	0.01 [-0.03, 0.04]	-0.01 [-0.05, 0.02]	0.29 [0.07, 0.52]	0.12	.005	.02
MFG_HbR_BA	-0.01 [-0.02, 0.00]	-0.02 [-0.03, -0.01]	-0.07 [-0.32, 0.16]	0.12	.27	.36
SMA_HbR_BA	-0.03 [-0.05, -0.02]	-0.02 [-0.04, 0.00]	-0.12 [-0.37, 0.14]	0.13	.18	.31
SMA_HbR_SC	-0.04 [-0.06, -0.02]	-0.03 [-0.04, -0.01]	-0.07 [-0.33, 0.18]	0.13	.31	.36

SFGmedial_HbO_BA	0.01 [-0.04, 0.07]	-0.06 [-0.11, 0.00]	0.24 [0.02, 0.46]	0.12	.02	.06
SFGmedial_HbO_SC	0.01 [-0.04, 0.06]	-0.04 [-0.09, 0.01]	0.33 [0.10, 0.55]	0.12	.003	.02
PoCG_HbO_SC	0.08 [0.02, 0.13]	0.13 [0.07, 0.19]	-0.11 [-0.35, 0.14]	0.12	.20	.31
PoCG_HbR_BA	-0.02 [-0.03, -0.01]	-0.03 [-0.04, -0.02]	0.02 [-0.23, 0.25]	0.12	.42	.45
PoCG_HbR_SC	-0.03 [-0.05, -0.02]	-0.05 [-0.07, -0.04]	0.14 [-0.11, 0.37]	0.12	.14	.31

Relationship between hemodynamics and balance performance (moderated regression)

Supplementary Table 4. Robust moderated regression analysis (Yohai, 1987; Koller and Stahel, 2017) of balancing-induced hemodynamics as predictor of wobble board performance (CoP path length), corrected for the influence of age group, sex and Baecke's sport score. Note that an age group-by-hemodynamics interaction term was also modelled. Results from simple slopes analysis shown under β_{old} and β_{young}, $\beta_{interaction}$ denotes results for the interaction. Standard errors (and associated test statistics and *p*-values) and confidence intervals were estimated based on *B* = 10000 bootstrap replications (Salibián-Barrera et al., 2008). Multiple testing correction based on the false discovery rate (FDR) was applied according to the procedure proposed by Benjamini and Hochberg (1995). Abbreviations as in Supplementary Table 1.

	**Simple slope "old"**				**Simple slope "young"**				**Interaction (difference in skopes)**			
ROI	β_{old}	SE	*p* (unc.)	*p* (FDR-corr.)	β_{young}	SE	*p* (unc.)	*p* (FDR-corr.)	$\beta_{interaction}$	SE	*p* (unc.)	*p* (FDR-corr.)
PreCG_HbR_BA	-0.07<br>[-0.30, 0.16]	0.12	.27	.31	-0.11<br>[-0.33, 0.10]	0.11	.16	.47	-0.04<br>[-0.35, 0.28]	0.16	.40	.40
PreCG_HbR_SC	-0.05<br>[-0.29, 0.17]	0.12	.33	.33	0.04<br>[-0.19, 0.26]	0.12	.37	.47	0.09<br>[-0.23, 0.42]	0.17	.29	.37
SFG_HbO_BA	0.22<br>[-0.02, 0.47]	0.13	.04	.08	-0.01<br>[-0.22, 0.20]	0.11	.47	.47	-0.23<br>[-0.55, 0.09]	0.16	.08	.18
SFG_HbO_SC	0.36<br>[0.12, 0.59]	0.12	.002	.01	-0.02<br>[-0.23, 0.18]	0.11	.43	.47	-0.38<br>[-0.68, -0.07]	0.16	.008	.05
SFG_HbR_BA	-0.36<br>[-0.62, -0.11]	0.13	.004	.01	-0.08<br>[-0.27, 0.10]	0.09	.20	.47	0.29<br>[-0.04, 0.61]	0.16	.04	.14
MFG_HbO_BA	0.29<br>[0.08, 0.53],	0.11	.004	.01	0.05<br>[-0.16, 0.26]	0.10	.31	.47	-0.24<br>[-0.55, 0.05]	0.15	.051	.14
MFG_HbR_BA	-0.33	0.16	.003	.01	-0.16	0.10	.052	.47	0.17	0.16	.15	.21

	[-0.57, -0.09]				[-0.37, -0.03]				[-0.14, 0.48]			
SMA_HbR_BA	-0.16<br>[-0.47, 0.16]	0.16	.16	.22	0.04<br>[-0.16, 0.24]	0.10	.33	.47	0.20<br>[-0.16, 0.56]	0.18	.14	.21
SMA_HbR_SC	-0.16<br>[-0.44, 0.13]	0.15	.14	.22	0.08<br>[-0.13, 0.27]	0.10	.24	.47	0.24<br>[-0.12, 0.57]	0.17	.09	.18
SFGmedial_HbO_BA	0.18<br>[-0.06, 0.41]	0.12	.07	.12	-0.02<br>[-0.23, 0.20]	0.11	.43	.47	-0.20<br>[-0.51, 0.12]	0.16	.11	.19
SFGmedial_HbO_SC	0.48<br>[0.25, 0.71]	0.10	.0001	.001	-0.05<br>[-0.24, 0.15]	0.10	.32	.47	-0.53<br>[-0.83, -0.23]	0.15	.0003	.004
PoCG_HbO_SC	0.25<br>[0.01, 0.49]	0.12	.02	.05	-0.08<br>[-0.29, 0.14]	0.11	.22	.47	-0.33<br>[-0.65, 0]	0.16	.02	.12
PoCG_HbR_BA	-0.10<br>[-0.33, 0.12]	0.11	.20	.25	-0.15<br>[-0.37, 0.07]	0.12	.10	.47	-0.05<br>[-0.37, 0.26]	0.16	.38	.40
PoCG_HbR_SC	-0.06<br>[-0.32, 0.17]	0.12	.30	.32	0.01<br>[-0.21, 0.22]	0.11	.46	.47	0.07<br>[-0.25, 0.40]	0.17	.33	.38

Hemodynamics as mediator in the relationship between age and balance performance

Supplementary Table 5. Robust mediation analysis (Zu and Yuan, 2010) addressing whether the relationship between age group and balance performance would be conveyed via balancing-induced hemodynamics. Analyses were conducted using residualized scores of the mediator and the criterion, i.e., the influence of sex and Baecke's sport score was partialled out before the analysis. A Huber-type weight to downweight extreme cases was applied ($\kappa = 0.05$) as proposed by Zu and Yuan (2010). Note that none of the results presented below would survive correction for multiple comparisons (all FDR-corr. p's > .05).

ROI	*a*	*b*	*ab* (95% CI)	*p*
PreCG_HbR_BA	-0.20	-0.09	0.02 [-0.04, 0.09]	.65
PreCG_HbR_SC	-0.14	0.05	-0.01 [-0.08, 0.03]	.73
SFG_HbO_BA	-0.40	0.10	-0.04 [-0.17, 0.02]	.23
SFG_HbO_SC	-0.52	0.18	-0.09 [-0.22, 0]	.04
SFG_HbR_BA	0.03	-0.21	-0.01 [-0.12, 0.10]	.90
MFG_HbO_BA	-0.31	0.16	-0.05 [-0.20, 0.02]	.25
MFG_HbR_BA	-0.27	-0.18	0.05 [-0.06, 0.19]	.33
SMA_HbR_BA	0.23	-0.03	-0.01 [-0.07, 0.07]	.88
SMA_HbR_SC	0.28	0.01	0.004 [-0.07, 0.09]	.94
SFGmedial_HbO_BA	-0.51	0.11	-0.06 [-0.21, 0.02]	.17
SFGmedial_HbO_SC	-0.49	0.19	-0.09 [-0.24, 0.01]	.07
PoCG_HbO_SC	0.43	0.09	0.04 [-0.03, 0.14]	.35

PoCG_HbR_BA	-0.38	-0.11	0.04 [-0.04, 0.16]	.33
PoCG_HbR_SC	-0.38	0.02	-0.01 [-0.11, 0.05]	.79

REFERENCES

Baldwin, S. A., Murray, D. M., and Shadish, W. R. (2005). Empirically supported treatments or type I errors? Problems with the analysis of data from group-administered treatments. *J Consult Clin Psychol* 73, 924–935. doi: 10.1037/0022-006X.73.5.924

Benjamini, Y., and Hochberg, Y. (1995). Controlling the False Discovery Rate: A Practical and Powerful Approach to Multiple Testing. *J R Stat Soc Series B Stat Methodol* 57, 289–300. doi: 10.1111/j.2517-6161.1995.tb02031.x

Cicchetti, D. V. (1994). Guidelines, criteria, and rules of thumb for evaluating normed and standardized assessment instruments in psychology. *Psychol Assess* 6, 284–290. doi: 10.1037/1040-3590.6.4.284

Cohen, J. (1988). *Statistical power analysis for the behavioral sciences*. Hillsdale, NJ: Erlbaum.

Fleiss, J. L. (1986). *The Design and Analysis of Clinical Experiments*. New York, NY: Wiley.

Koller, M., and Stahel, W. A. (2017). Nonsingular subsampling for regression S estimators with categorical predictors. *Comput Stat* 32, 631–646. doi: 10.1007/s00180-016-0679-x

McGraw, K. O., and Wong, S. P. (1996). Forming inferences about some intraclass correlation coefficients. *Psychol Methods* 1, 30–46. doi: 10.1037/1082-989X.1.1.30

Salibián-Barrera, M., van Aelst, S., and Willems, G. (2008). Fast and robust bootstrap. *Stat Methods Appt* 17, 41–71. doi: 10.1007/s10260-007-0048-6

Yohai, V. J. (1987). High Breakdown-Point and High Efficiency Robust Estimates for Regression. *Ann. Statist.* 15, 642–656. doi: 10.1214/aos/1176350366

Zu, J., and Yuan, K.-H. (2010). Local Influence and Robust Procedures for Mediation Analysis. *Multivariate Behav Res* 45, 1–44. doi: 10.1080/00273170903504695
